# Efficacy of Co-Trimoxazole against Experimental Melioidosis Acquired by Different Routes of Infection

**DOI:** 10.1128/aac.00708-22

**Published:** 2022-10-13

**Authors:** Michelle Nelson, Neil Burton, Alejandro Nunez, Wendy Butcher, Sarah Ngugi, Timothy P. Atkins

**Affiliations:** a CBR Division, Defence Science and Technology Laboratory (Dstl), Porton Down, Salisbury, Wiltshire, United Kingdom; b Q3 Analytical Ltd., Porton Science Park, Porton Down, Salisbury, United Kingdom; c Animal and Plant Health Agency, Weybridge, Addlestone, Surrey, United Kingdom

**Keywords:** animal model, marmoset, melioidosis, memory T cell, relapse

## Abstract

Burkholderia pseudomallei is the causative agent of melioidosis and presents with diverse clinical manifestations. Naturally occurring infection occurs following contamination of cuts or skin abrasions, or ingestion of contaminated water, and occasionally through inhalational of infected soil or water particles. The influence of the route of disease acquisition on the efficacy of medical countermeasures has not been explored in humans or in appropriate animal models. The efficacy of co-trimoxazole against melioidosis acquired by different routes of exposure was assessed in postexposure prophylaxis (PEP) and treatment studies in marmoset models of melioidosis. Following challenge with B. pseudomallei by the inhalational, subcutaneous, or ingestion routes of administration, animals were given co-trimoxazole at 12 hourly intervals for 14 days, starting either 6 h postchallenge or at the onset of fever. Animals were then observed for 28 days. All animals that received antibiotic 6 h postchallenge survived the duration of dosing. All animals that received antibiotics at the onset of fever completed the treatment, but 10%, 57%, and 60% of those with ingestion, subcutaneous, and inhalation challenge relapsed, respectively. Bacteriological and histological differences were observed between placebo-control animals and those that relapsed. Immunological profiles indicate difference between animals given placebo and those that relapsed or survived the duration of the study. A broad T-cell activation was observed in animals that survived. Overall, these data suggest the efficacy of co-trimoxazole, as measured in the incidence of relapse, differs depending on the disease-acquisition route. Therefore, there are implications in treating this disease in regions of endemicity.

## INTRODUCTION

Melioidosis is an emerging infectious disease that is endemic to many tropical and subtropical regions, most notably southeast Asia and northern Australia (1). It is caused by the Gram-negative, facultative intracellular bacterium Burkholderia pseudomallei. There are three naturally occurring routes of infection; cutaneous, inhalational, or ingestion (2). Activities that involve exposure to infected water or soil have been associated with increased likelihood of acquiring disease (2). These studies also showed that an individual was twice as likely to have melioidosis in areas with bacteria in the drinking water as in areas without contaminated water supplies. This indicates that ingestion is likely to be an important route of human B. pseudomallei infection. However, the route of infection is often unclear from the clinical presentation of the disease.

B. pseudomallei is inherently resistant to a number of antibiotics, making treatment difficult. Treatment is undertaken in two stages: the initial acute phase treatment and the prolonged eradication phase. Intravenous ceftazidime or meropenem are the recommended antibiotics for the intensive acute phase, with oral co-trimoxazole the recommended antibiotic for the eradication phase (3, 4). A clinical trial showed that co-trimoxazole on its own was equally as effective as co-trimoxazole and doxycycline during the eradication phase (5). Oral co-trimoxazole has also been recommended as a prophylactic agent (3) and was subsequently recommended prophylactically in hemodialysis patients (6); however, the practicalities of adopting this approach universally have been challenged (7). Murine studies have shown co-trimoxazole to be efficacious for prophylaxis for inhalational melioidosis (8, 9). However, the influence of the route of disease acquisition on the efficacy of medical countermeasures has not been explored. This, of course, has clinical implications for treating individuals but also when performing clinical trials to assess novel or alternative antimicrobial products.

The common marmoset (Callithrix jacchus) has been used to characterize a number of infectious diseases, including melioidosis. Indeed, marmoset models have been established for all three naturally occurring routes of melioidosis (10 to 12). In all three models, melioidosis is characterized by an acute, febrile, lethal disease, although there are some subtle differences associated with the route of acquisition. Pneumonia is observed in all marmosets challenged by the inhalational route, although it only occurs in less than 20% of marmosets challenged by either the ingestion or subcutaneous routes. Skin lesions are only observed following subcutaneous challenge, and lesions in the gastrointestinal tract are only observed following ingestion challenge. These differences are sufficient to distinguish between the routes of challenge in the animal model and may be useful to understand differences in the efficacy of medical countermeasures.

The aim of this study was to explore the influence of the route of disease acquisition on the efficacy of a test antibiotic, co-trimoxazole, in both prophylaxis and treatment studies.

## RESULTS

### Pharmacokinetic studies.

In order to determine a human-equivalent dose of co-trimoxazole in the marmoset, two studies were performed: a single-dose study and a multiple-dose study.

**FIG 1:**
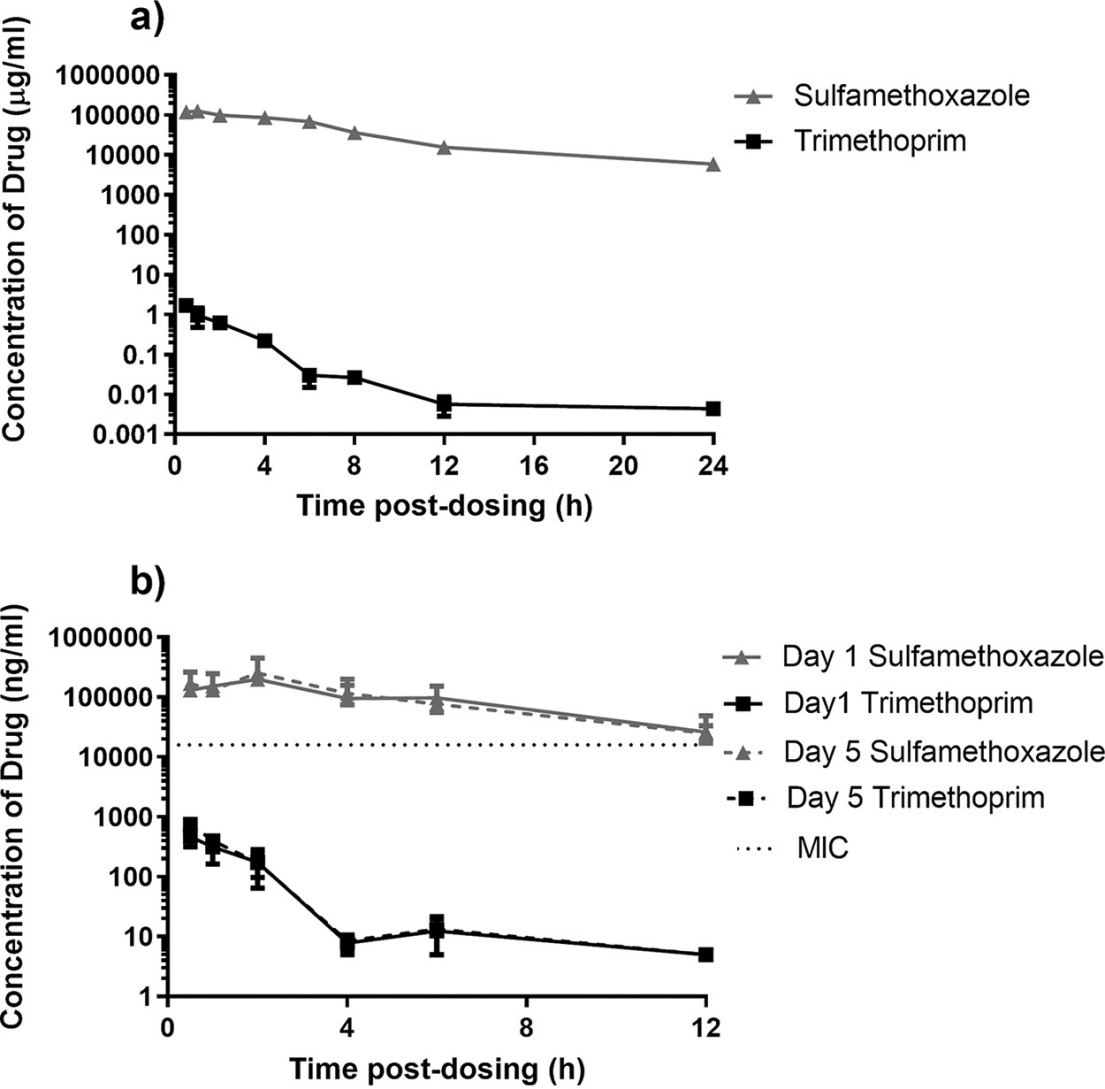
Concentration of antibiotic in the plasma of marmosets following administration of co-trimoxazole. (a) Single oral administration of 13 mg/kg of trimethoprim and 66 mg/kg of sulfamethoxazole (a total of 79 mg/kg of antibiotic). (b) Multiple 12 hourly oral administration of 79 mg/kg of antibiotic with blood collected following dose 1 (day 1) or dose 9 (day 5).

**(i) Single-dose study.** The concentration of the two components of co-trimoxazole, trimethoprim and sulfamethoxazole, were assessed in the plasma of marmosets periodically following oral administration of 13 mg/kg of trimethoprim and 66 mg/kg of sulfamethoxazole (a total of 79 mg/kg of antibiotic) (Fig. 1a). A maximum concentration (Cmax) of 143 μg/mL and 1.7 μg/mL was reached by 1 and 0.5 h (T_max_) for sulfamethoxazole and trimethoprim, respectively. Reasonable levels of sulfamethoxazole (5.8 μg ± 2.6 μg) were detected in the plasma at 24 h postdosing. However, there was only a low level of trimethoprim (4 ± 0.6 ng). The half-life (t_1/2_) of these components was 5.1 and 2.9 h for sulfamethoxazole and trimethoprim, respectively.

The pharmacokinetic parameters obtained following a single oral dose of 79 mg/kg of co-trimoxazole were used to perform nonparametric superposition analysis in Phoenix WinNonLin (Pharsight v 6.1). For a single oral administration of co-trimoxazole, the concentration of sulfamethoxazole is above the MIC (16 μg/mL) for 49% of the time in a 24-h period. Increasing the frequency of administration to every 12 h increases the time above the MIC to 99.7%. However, the time above the MIC (32 μg/mL) for trimethoprim for both of these regimens is 0%.

**(ii) Multidose study.** The concentration of antibiotic components in the plasma of marmosets was analyzed periodically following oral administration of 79 mg/kg of co-trimoxazole (Fig. 1b). A T_max_ of 2 h for the sulfamethoxazole component of co-trimoxazole was the same following a single dose and following 9 doses. The Cmax increased slightly after dose 9 to 252 μg/mL compared to 198 μg/mL after a single dose. The t_1/2_ also increased slightly from 3.25 to 3.65 h. A similar pattern was observed for trimethoprim, with a T_max_ of 0.5 h and an increase in the Cmax from 198 μg/mL to 252 μg/mL following doses 1 and 9, respectively. However, the t_1/2_ decreased slightly from 1.3 to 1.2 h, respectively.

### Efficacy studies.

**(i) Postexposure prophylaxis (PEP).** As a proof-of-concept study, four marmosets were challenged with B. pseudomallei by each of the inhalational, ingestion, and subcutaneous routes of challenge. The challenge dose was the lowest dose that was reproducibly lethal based on previous model development studies, such that the challenge dose was 1.37 × 10^2^ ± 18, 1.16 × 10^2^, and 1.2 × 10^7^ CFU for the inhalational, subcutaneous, and ingestion routes, respectively. At 6.0 ± 0.2 h postchallenge, half of the animals were given 79 mg/kg of co-trimoxazole by the oral route, and the remaining animals received placebo. Dosing continued at 12 hourly intervals for 14 days. The animals were then observed for a further 14 days.

**FIG 2:**
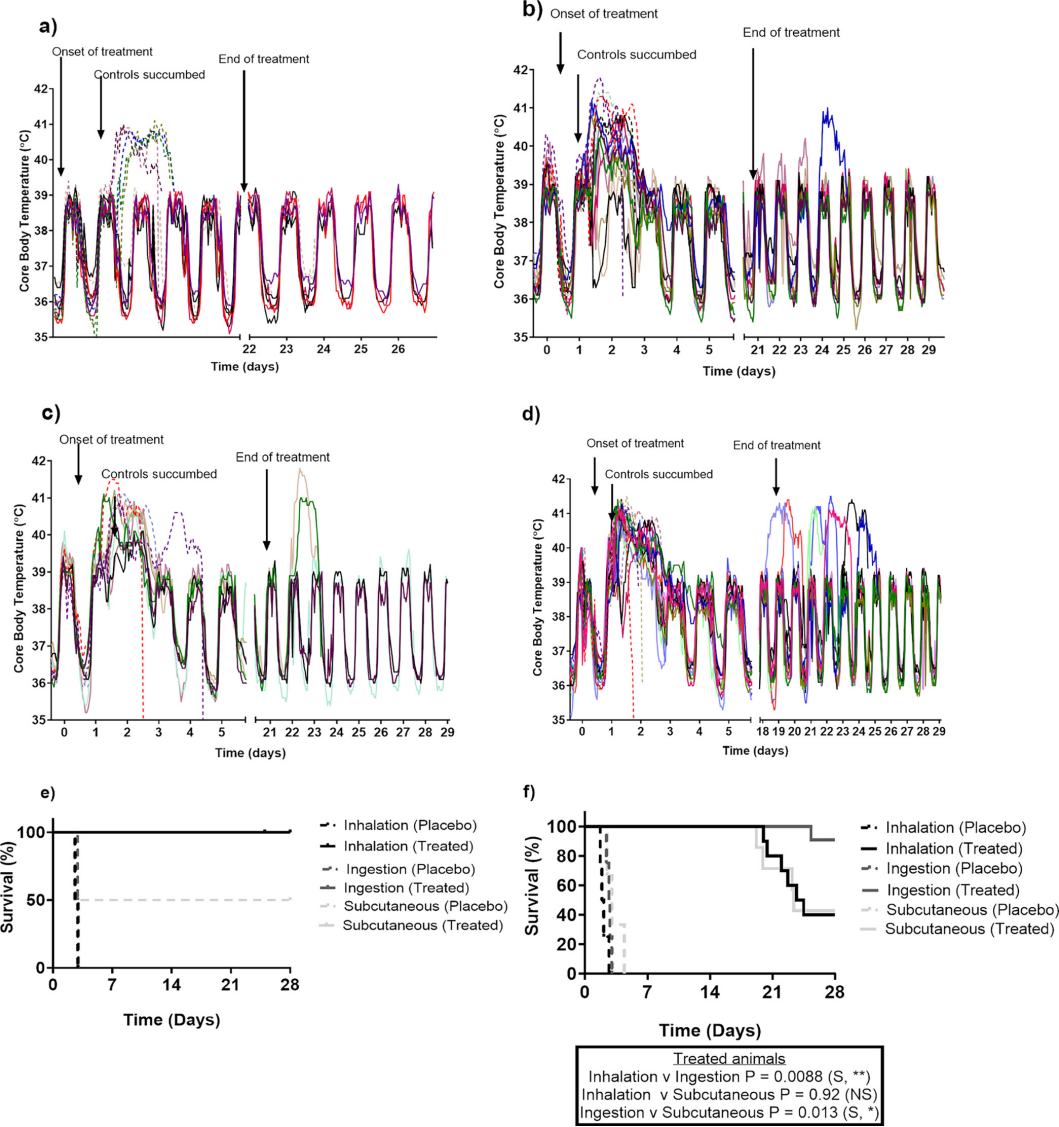
Temperature and survival of marmosets challenged with B. pseudomallei by various routes of infection. (a) All routes of infection following administration of co-trimoxazole 6 h postchallenge. (b) Ingestion route of infection following administration of co-trimoxazole at the onset of fever. (c) Subcutaneous route of infection following administration of co-trimoxazole at the onset of fever. (d) Inhalational route of infection following administration of co-trimoxazole at the onset of fever. (e) Survival following treatment initiation at 6 h postchallenge, prophylaxis. (f) Survival following treatment initiation at the onset of fever, trigger-to-treat.

All animals maintained their normal diurnal rhythm for between 27 and 40 h postchallenge when the placebo-treated animals became febrile (defined as greater than 40°C for at least 3 consecutive readings) (Fig. 2a). Animals that received placebo became febrile at a mean time of 33.1 ± 5.4 h postchallenge; 27.7 and 35.7 h postchallenge following inhalational challenge; and 34.8 and 39.8 h postchallenge following ingestion challenge. Only one placebo animal challenged by the subcutaneous route became febrile at 27.4 h postchallenge. Animals then remained febrile for 33.0 ± 2.9 h postchallenge prior to reaching the humane endpoint. The febrile response was present prior to the onset of clinical signs. Clinical signs were first observed between 42 and 46 h postchallenge for animals challenged by the ingested and inhalational routes, respectively. The clinical signs initially presented as an unkempt coat, reluctance to move, and a change in behavior with the animals becoming more subdued. Clinical signs progressed within 24 h when animals became more subdued and somnolent prior to reaching the humane endpoint by 74 h postchallenge. One placebo-treated animal challenged by the subcutaneous route became subdued approximately 6 h following the onset of fever and became slightly hunched 24 h later and reached the humane endpoint at 59 h postchallenge.

There was no febrile response or clinical signs observed in any animal that was administered co-trimoxazole, and they all survived until the end of the study (Fig. 2e). However, one animal that received placebo following challenge by the subcutaneous route also survived for the duration of the study. No bacteria were recovered from any of the animals that survived the duration of the study, they were immunologically indistinct from naive animals, and no histopathological changes in cellular morphology were observed. Due to the low number of animals, there was no statistical significance in the survival of placebo and co-trimoxazole-treated animals for any routes of challenge. This study provided evidence that co-trimoxazole therapy was effective for melioidosis in the marmoset, and the remaining studies focused on assessing temperature as a trigger-to-treat.

### Treatment.

Fourteen animals were challenged by the inhalational and ingestion route of challenge, and 10 animals were challenged by the subcutaneous route (Table 1). The challenge dose was the lowest dose that was reproducibly lethal based on previous model development studies, such that the challenge dose was 5.03 × 10^2^ ± 1.3 × 10^2^; 5.76 × 10^1^ ± 7; and 5.98 × 10^6^ ± 7.4 × 10^5^ CFU for the inhalational, subcutaneous, and ingestion routes, respectively. At the onset of fever, 10 animals that were challenged by the inhalational and ingestion route, and 7 animals that were challenged by the subcutaneous route, were administered 79 mg/kg of co-trimoxazole by the oral route. The remaining animals received placebo. Co-trimoxazole was administered at 31.8 ± 4.9 h following challenge and 2.2 ± 1.2 h following fever (Fig. 2c, d and e). There was no correlation between the time of the first dose following fever and whether the animals survived the duration of the study or relapsed following cessation of antibiotic. The second antibiotic dose was administered within 12 h of the initial dose, and then animals were dosed at 12 hourly intervals for a total of 28 doses.

**TABLE 1:** Animal summary data for animals challenged with *B. pseuodmallei* by various routes and administered co-trimoxazole at the onset of fever^*a*^

Animal ID	Challenge route	Test article/time of treatment	Time to onset of fever (h)	Duration of fever (h)	Bacteriology at onset of fever (CFU/mL)	Outcome
109W	Inhalational	Co-trimoxazole	21.4	39.1	30	Relapsed
73W	Inhalational	Co-trimoxazole	21.1	47.9	8	Survived
112X	Inhalational	Co-trimoxazole	21.2	36.6	10	Relapsed
94X	Inhalational	Co-trimoxazole	22.0	26.5	0	Relapsed
126X	Inhalational	Co-trimoxazole	20.7	41.9	15	Survived
117X	Inhalational	Co-trimoxazole	23.3	35.0	25	Relapsed
40X	Inhalational	Co-trimoxazole	22.3	43.7	N/A	Survived
77X	Inhalational	Co-trimoxazole	23.8	42.7	0	Relapsed
63Y	Inhalational	Co-trimoxazole	24.7	42.0	10	Survived
61Y	Inhalational	Co-trimoxazole	24.2	41.9	10	Relapsed
38X	Inhalational	Placebo	21.9	23.6	0	Succumbed
13X	Inhalational	Placebo	21.0	16.5	5	Succumbed
75X	Inhalational	Placebo	21.3	27.7	10	Succumbed
21Y	Inhalational	Placebo	30.0	33.7	N/A	Succumbed
55W	Subcutaneous	Co-trimoxazole	33.3	36.8	N/A	Survived
57X	Subcutaneous	Co-trimoxazole	26.2	43.8	IS	Relapsed
86Y	Subcutaneous	Co-trimoxazole	34.0	35.5	25	Relapsed
152X	Subcutaneous	Co-trimoxazole	33.6	34.0	N/A	Survived
133Y	Subcutaneous	Co-trimoxazole	34.9	37.7	20	Relapsed
11Y	Subcutaneous	Co-trimoxazole	32.8	36.5	10	Survived
36Y	Subcutaneous	Co-trimoxazole	33.3	35.8	N/A	Relapsed
112W	Subcutaneous	Placebo	32.9	73.3	0	Succumbed
115W	Subcutaneous	Placebo	27.6	32.9	8	Succumbed
103Y	Subcutaneous	Placebo	34.2	37.3	0	Succumbed
127W	Ingestion	Co-trimoxazole	32.7	35.6	0	Survived
152W	Ingestion	Co-trimoxazole	34.8	28.4	N/A	Survived
47X	Ingestion	Co-trimoxazole	28.7	40.8	10	Relapsed
87X	Ingestion	Co-trimoxazole	29.9	29.0	0	Survived
124W	Ingestion	Co-trimoxazole	36.0	35.5	N/A	Survived
70X	Ingestion	Co-trimoxazole	36.0	33.9	N/A	Survived
90X	Ingestion	Co-trimoxazole	36.7	1.0	N/A	Survived
33Y	Ingestion	Co-trimoxazole	36.5	33.2	0	Survived
45Y	Ingestion	Co-trimoxazole	37.0	33.7	N/A	Survived
98Y	Ingestion	Co-trimoxazole	35.4	34.3	20	Survived
122W	Ingestion	Placebo	30.4	27.0	15	Succumbed
9X	Ingestion	Placebo	31.6	38.0	4	Succumbed
105W	Ingestion	Placebo	34.1	30.4	0	Succumbed
139W	Ingestion	Placebo	33.8	31.3	10	Succumbed

a N/A, not applicable; IS, insufficient sample.

### Clinical picture at the onset of fever.

**FIG 3:**
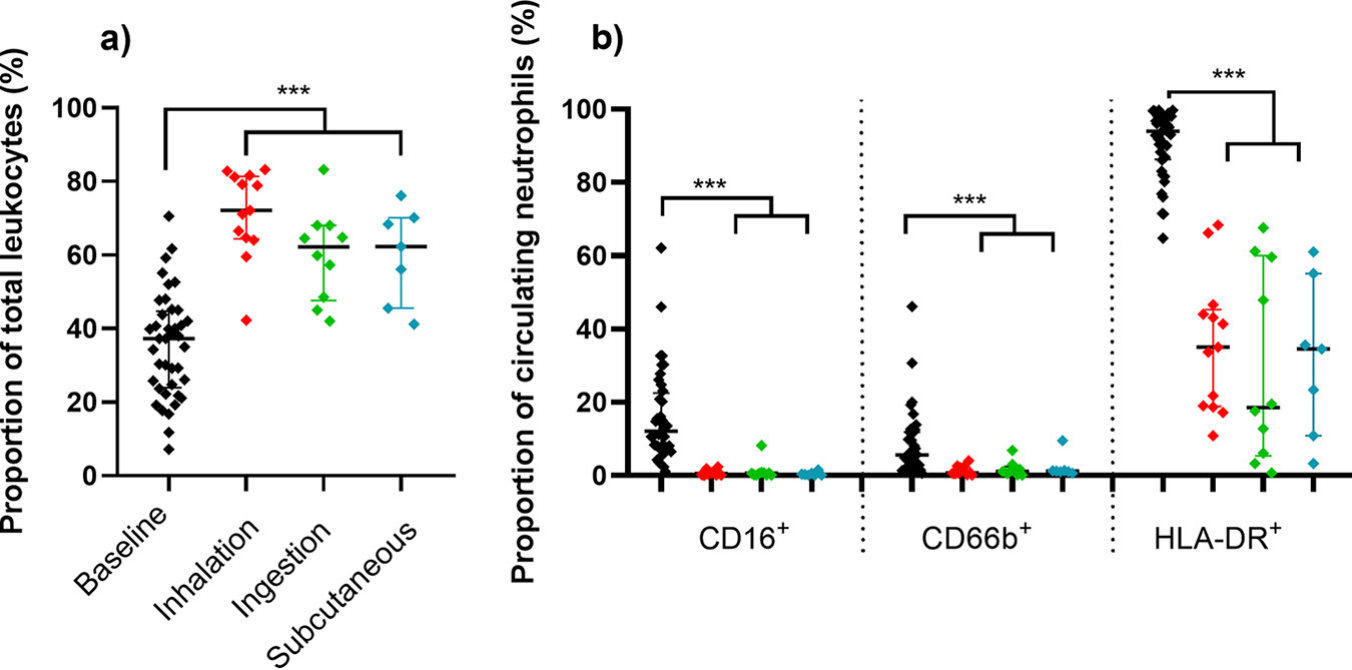
Changes in the proportion of circulating neutrophils and expression of activation markers at the onset of fever. Data are presented as median values with the interquartile range displayed. The significant difference was determined by Kruskal-Wallis ANOVA, where ***, *P* < 0.001.

There was a significant difference in the time to the onset of fever associated with the route of challenge: fever following inhalation challenge occurred significantly earlier at 22.8 ± 2.4 h than fever following subcutaneous challenge at 32.3 ± 2.9 h (*P* = 0.0071) or ingestion challenge at 33.3 ± 2.7 h (*P* = 0.0007). Prior to the initiation of treatment, blood was collected from animals to assess levels of bacteremia and assess the immunological response. Overall, 67% of animals were bacteremic with 12.9 ± 1.6 CFU/mL of blood. A higher, but nonsignificant, proportion of animals that were challenged by the inhalational route had bacteria in their blood (75%) compared with 67% of animals challenged by the subcutaneous route and 56% of those challenged by the ingestion route. Immunologically, levels of circulating neutrophils and their HLA-DR^+^ expression were assessed as key characteristics of acute melioidosis in the marmoset (measurable in a single blood sample) (13) (Fig. 3a). There were no challenge route-related differences in the response at this time. There was a significant increase in the proportion of neutrophils above baseline levels (baseline median 37% versus 65% at onset of fever, *P* < 0.001), and all animals had at least a 15% reduction in HLA-DR^+^ marker (94% versus 34%, *P* < 0.001) (Fig. 3b). The proportions of additional markers that are expressed on mature human neutrophils, CD16^+^ and CD66b^+^, were significantly reduced (12.1% versus 0.45% and 5.6% versus 1.05%, respectively, *P* < 0.001 for both), and levels of IFN-γ were above baseline levels in 10 out of 11 animals assessed (data not shown).

### Disease progression.

All animals administered co-trimoxazole at the onset of fever survived the duration of treatment. Fever was maintained for a mean time of 35.1 ± 10.4 h following the onset until animals either reached the humane endpoint (placebo controls) or resolved the fever (co-trimoxazole treated). There were no significant differences in the duration of fever and the route of challenge (39.7 ± 5.9, 30.5 ± 10.9, and 37.2 ± 3.2 h for inhalational, ingestion, and subcutaneous, respectively) (Fig. 2c, d, and e). There was also no significant difference in the duration of fever between placebo- and co-trimoxazole-treated animals following subcutaneous and ingestions challenge. However, the fever was significantly shorter in the placebo animals challenged by the inhalational route compared to those that received antibiotic and resolved fever (25.4 ± 3.6 CFU/mL versus 39.7 ± 1.9 CFU/mL, *P* = 0.0024).

Clinical signs were apparent in placebo-treated animals between 24 and 72 h postchallenge regardless of the route of infection. However, from 4 days following the cessation of treatment, 1 out 10 (10%) treated animals challenged by the ingestion route relapsed compared to 6 out of 10 (60%) or 4 out of 7 (57%) by the inhalational and subcutaneous routes, respectively (Fig. 2f). Animals that relapsed became febrile and reached the humane endpoint between 2 and 5 days postfever. All other animals remained normal. Therefore, there was a significant increase in survival following the administration of co-trimoxazole (40% versus 0%, *P* < 0.0001 [inhalation]; 43% versus 0%, *P* < 0.0001 [subcutaneous]; and 90% versus 0%, *P* = 0.0008 [ingestion]). However, the incidence of relapse was only significantly different for animals challenged by the ingestion route compared to both the inhalational and subcutaneous route (10% versus 60%, *P* = 0.0088, and 10% versus 57%, *P* = 0.013, respectively) (Fig. 2f). No statistical difference was observed between the incidence of relapse for the inhalational and subcutaneous routes (60% versus 57%, *P* = 0.92). There was also no significant difference in the time to relapse associated with the route of challenge, with a median time of 22.4, 23.2, and 25.3 days for the inhalational, subcutaneous, and ingestion routes, respectively.

### Bacterial load.

Levels of bacterial colonization of the liver, spleen, kidneys, lungs, and blood were assessed at the time of postmortem by plate culture. No bacteria were recovered from any of the animals that survived the duration of the study in both the PEP and treatment studies. All animals that succumbed to challenge had high levels of bacteria in all organ types assessed, and in the treatment study this was regardless of whether they had received placebo or relapsed following the cessation of co-trimoxazole (Table 2). A difference in the bacterial load was observed in animals challenged by the inhalational route, depending on whether they received no antibiotic (placebo) or they relapsed; significantly higher numbers of bacteria were present in the kidney and blood of animals that relapsed after the cessation of antibiotics (5.3 log_10_ CFU/g versus 4.0 log_10_ CFU/g, *P* = 0.0203, and 4.4 log_10_ CFU/g versus 3.3 log_10_ CFU/g, *P* = 0.0479, respectively). This was associated with a nonsignificant decrease in the number of bacteria recovered from the lungs of these animals (6.1 log_10_ CFU/g versus 7.1 log_10_ CFU/g, *P* = 0.0634). This difference was not observed in animals that were challenged by the ingested or subcutaneous routes.

**TABLE 2:** Comparison of the bacterial load from various marmoset tissues at the time of euthanasia following challenge with B. pseudomallei by various routes with or without administration of co-trimoxazole at the onset of fever^*a*^

Challenge route	Organ	Placebo	Treated-relapsed	Significance between placebo and relapsed groups	Significance between placebo controls of each challenge route
Inhalation	Liver	5.5 (4.3 to6.7)	6.6 (6.0 to7.3)	ns	ns
	Spleen	6.8 (6.1 to7.6)	7.6 (7.0 to8.2)	ns	ns
	Kidney	4.0 (2.9 to5.1)	5.3 (4.8 to5.9)	*P* = 0.0203	*P* = 0.0199 between inhalation and ingestion
	Lung	7.1 (6.1 to 8.0)	6.1 (5.6 to 6.8)	ns	*P* = 0.007 between inhalation and ingestion
	Blood	3.3 (2.3 to 4.3)	4.4 (3.7 to 5.1)	*P* = 0.0479	*P* = 0.015 between inhalation and ingestion
					*P* = 0.0179 between inhalation and subcutaneous
Subcutaneous	Liver	6.5 (5.6 to 7.5)	6.4 (5.2 to 7.5)	ns	
	Spleen	7.5 (7.0 to 7.9)	7.7 (7.7 to 7.8)	ns	
	Kidney	5.1 (4.4 to 5.7)	5.3 (3.8 to 6.8)	ns	
	Lung	6.0 (5.4 to 6.6)	5.9 (5.4 to 6.3)	ns	
	Blood	4.9 (4.2 to 5.6)	4.4 (3.3 to 5.5)	ns	

Ingestion	Liver	6.4 (5.8 to 7.1)	6.9	n/a	
	Spleen	6.8 (5.8 to 7.9)	7	n/a	
	Kidney	5.6 (4.6 to 6.6)	5.6	n/a	
	Lung	5.7 (5.2 to 6.2)	6.2	n/a	
	Blood	4.5 (4.3 to 4.8)	3.9	n/a	

a Bacterial load data are presented as log transformation of the number of CFU (CFU present with 95% confidence intervals). ns indicates nonsignificance, and n/a is not applicable due to there only being 1 animal that relapsed following ingestion challenge.

In the placebo control animals, there was no significant difference in the concentration of bacteria recovered in the liver and spleen of animals challenged by the inhalational, subcutaneous, or ingested route of challenge (Table 2). However, there were significantly lower concentrations of bacteria recovered from the kidneys and blood of animals challenged by the inhalational route compared to those challenged by the ingested route (4.0 log_10_ CFU/g versus 5.6 log_10_ CFU/g, *P* = 0.0199, and 3.3 log_10_ CFU/g versus 4.5 log_10_ CFU/g, *P* = 0.015, respectively). Additionally, there were significantly higher levels of bacteremia for animals challenged by the subcutaneous route compared to inhalational challenge (4.9 log_10_ CFU/g versus 3.3 log_10_ CFU/g, *P* = 0.0179). There were also significantly more bacteria in the lungs following inhalational challenge than following ingested challenge (7.1 log_10_ CFU/g versus 5.7 log_10_ CFU/g, *P* = 0.007).

### Histology, immunohistochemistry (IHC), and electron microscopy.

**FIG 4:**
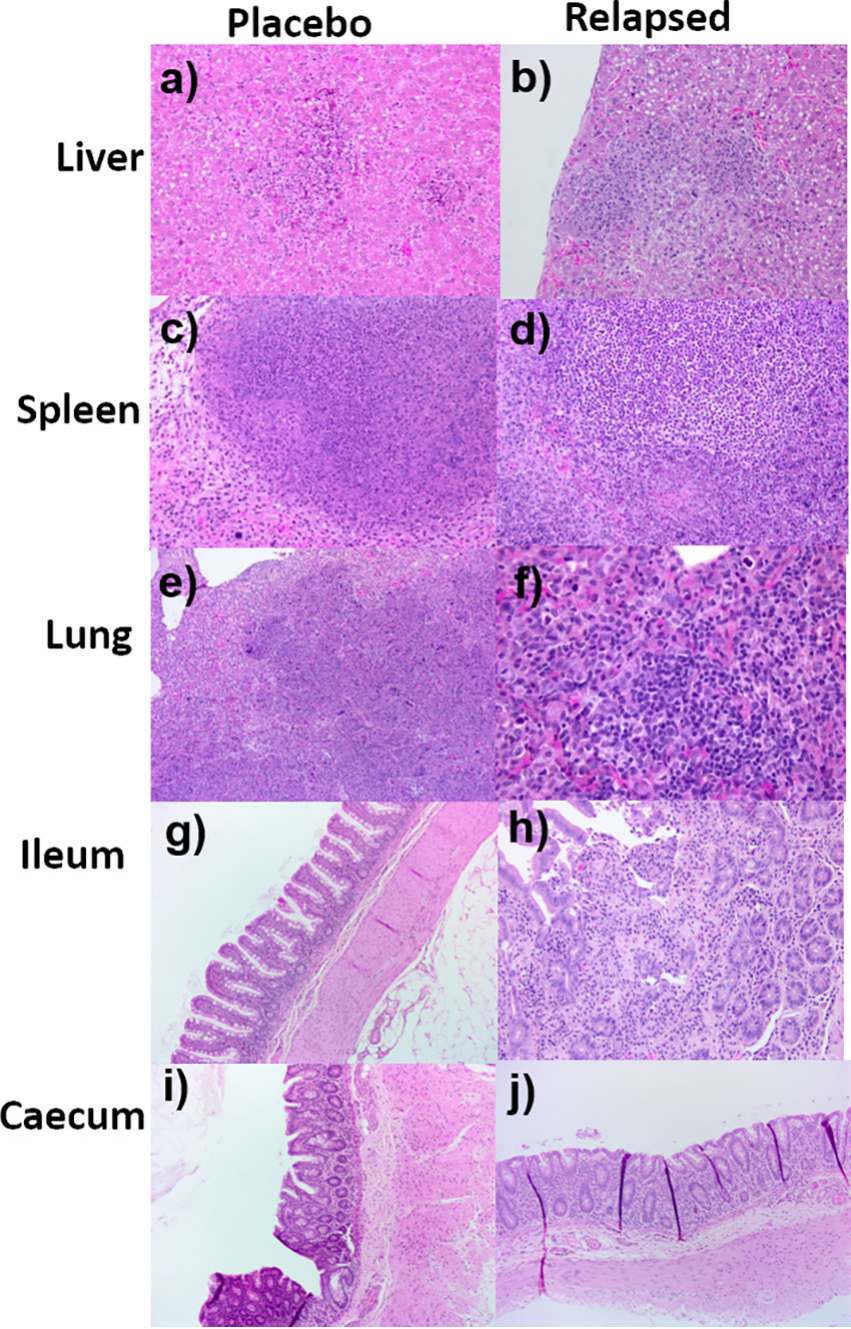
Representative H&E-stained tissue sections from marmosets’ challenge with B. pseudomallei by various routes of exposure; they were either given placebo or relapsed following treatment with co-trimoxazole. Liver sections from a placebo animal following ingestion challenge showing mild lesions (×20) (a), or an animal that relapsed following treatment with co-trimoxazole and inhalational challenge showing moderate lesions (×20) (b). Spleen sections showing moderate lesions from a placebo animal following ingestion challenge (c), and an animal that relapsed following treatment with co-trimoxazole and inhalational challenge (d) (×20). Lung sections from animals following inhalational challenge that were given either placebo and presented with severe lesions (e) or treatment with co-trimoxazole showing mild, chronic lesions (f). Ileum (g) and cecum (i) sections showing the lack of lesions in a placebo animal challenged by the inhalational route compared to the mild (h) or moderate (j) lesions observed in the ileum and cecum, respectively, of an animal that relapsed following treatment with co-trimoxazole and inhalational challenge.

Histological analysis indicates that all animals that succumbed to disease had mild to moderate melioidosis-associated random multifocal necrotizing hepatitis or splenitis regardless of the route of infection or whether they had relapsed following treatment or were placebo animals (Fig. 4). All animals that received placebo and succumbed following inhalational challenge had severe acute multifocal necrotizing interstitial pneumonia. However, all animals that relapsed following inhalational challenge had less severe or atypical pneumonia compared to placebo animals challenged by the inhalational route. Additionally, lesions were apparent in various parts of the gastrointestinal tract of these animals, which is atypical for this route of infection (Fig. 4). Animals that survived challenge with B. pseudomallei following administration of co-trimoxazole had no specific melioidosis-associated lesions, regardless of the route of infection.

**FIG 5:**
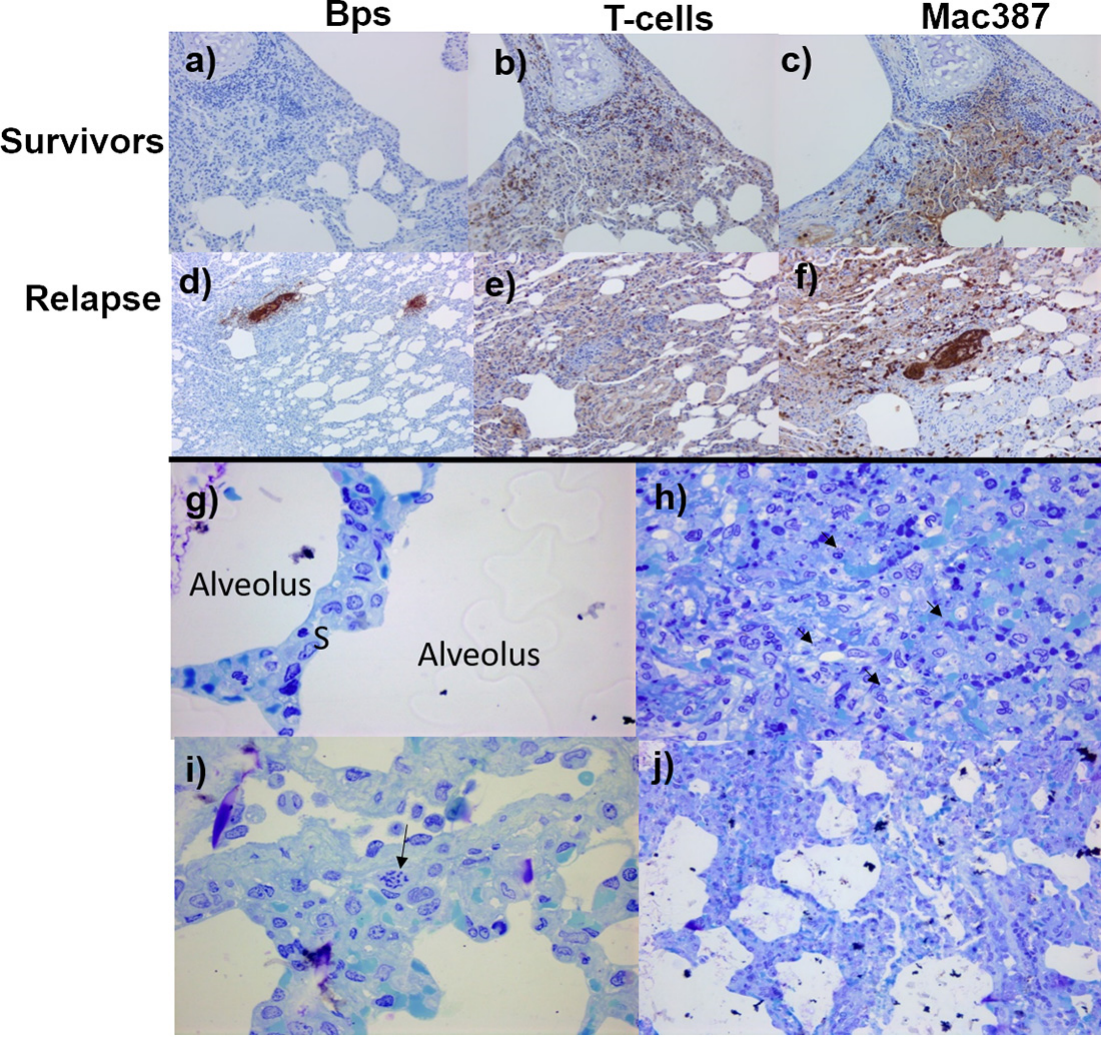
Representative lung section images from marmosets’ challenge with B. pseudomallei by the inhalational route and treated with co-trimoxazole at the onset of fever. IHC-stained sections from survivors (a to c) or relapsed following treatment (d to f). Sections were stained with an antibody to identify B. pseudomallei (Bps), indicating no presence of the bacteria in the lungs of survivors, but discrete foci of bacterial antigen staining in animals that relapsed (a and d). A similar, moderate amount of T-cell and macrophage/neutrophil staining observed in animals that survived or relapsed, with more intense macrophage/neutrophil staining located with B. pseudomallei antigen. Representative EM images (g, i, and j) are from the same animal that relapsed following treatment, indicating a region of normal pulmonary septum (g) (×1,000) and chronic changes indicated by alveolar septa thickened by histiocytic infiltration (arrow) (i) (×400). Evidence of intravascular bacteria, likely intracellular, were observed in an area of lung without significant changes supportive of hematogenous dissemination in relapsed animals (j). Acute lesion in lung from a placebo-treated animal showing alveolar exudation, cell debris, and loss of tissue structure with bacteria longitudinal sections in macrophages and extracellular locations (h).

To further characterize the lesions from animals with atypical lung lesions following challenge by the inhalational route, immunohistochemical staining was performed. The lesions were observed in all animals that were treated following inhalational challenge, regardless of whether they relapsed or survived. Levels of bacterial capsular antigen (Bps^+^), T cells (CD3^+^), and macrophages/neutrophils (MAC387^+^) were quantified in areas of interest (Fig. 5). The lesions were nonspecific, subacute to chronic interstitial pneumonia with occasional thickened septa. Five out the 6 animals that relapsed had minimal to low levels of bacterial antigen detected. This was multifocal to diffuse in areas of acute inflammatory exudation. The bacterial antigen extended into the alveoli and had multifocal intravascular staining that was probably intracellular but without significant histological changes. No staining was directly associated with the subacute to chronic lymphohistiocytic interstitial pneumonia. The highest levels of bacterial antigen observed in one animal was associated with moderate staining due to the presence of a thrombosis/embolism in a middle size vessel. There were moderate levels of CD3^+^ and MAC387^+^ staining in all animals whether they relapsed or survived. The increase in CD3^+^ occurred throughout the lung, particularly in the perivascular and peribronchial areas, in the alveolar septa, and in areas of septal thickening. For animals that survived, there was significant staining, especially in areas of lymphohistiocytic infiltration. MAC387 staining was also broadly distributed throughout the alveolar septa and typically more abundant than CD3 staining, whereas staining levels were much lower with MAC387 in the lymphoplasmacytic perivascular area and peribronchial infiltration. In the animals that survived, there was significant staining in both thickened and nonthickened septa, with occasional clustering or groupings identified by both hematoxylin and eosin (H&E) and electron microscopy (EM) (Fig. 5). Comparison of the acute and chronic lesions by EM show the presence of bacteria, although for the chronic lesions these are associated with less structural changes to the tissue (Fig. 5j).

The lesions in the gastrointestinal tract of animals that relapsed following inhalational challenge and treatment with co-trimoxazole were also assessed by IHC. These lesions were not observed in animals that succumbed to the initial challenge (placebo) or indeed in animals that survived following treatment. They were associated with positive antigen staining in lesions in the submucosa with lymphatic and vascular involvement. There was a moderate increase in the CD3^+^ staining in the lamina propria and in the submucosa around the lesion. Marked MAC387^+^ staining was observed in areas of exudation in submucosa and in the peritoneum. Low levels of scattered MAC387^+^ staining were observed in the lamina propria.

### Immunological profile in placebo control animals.

**FIG 6:**
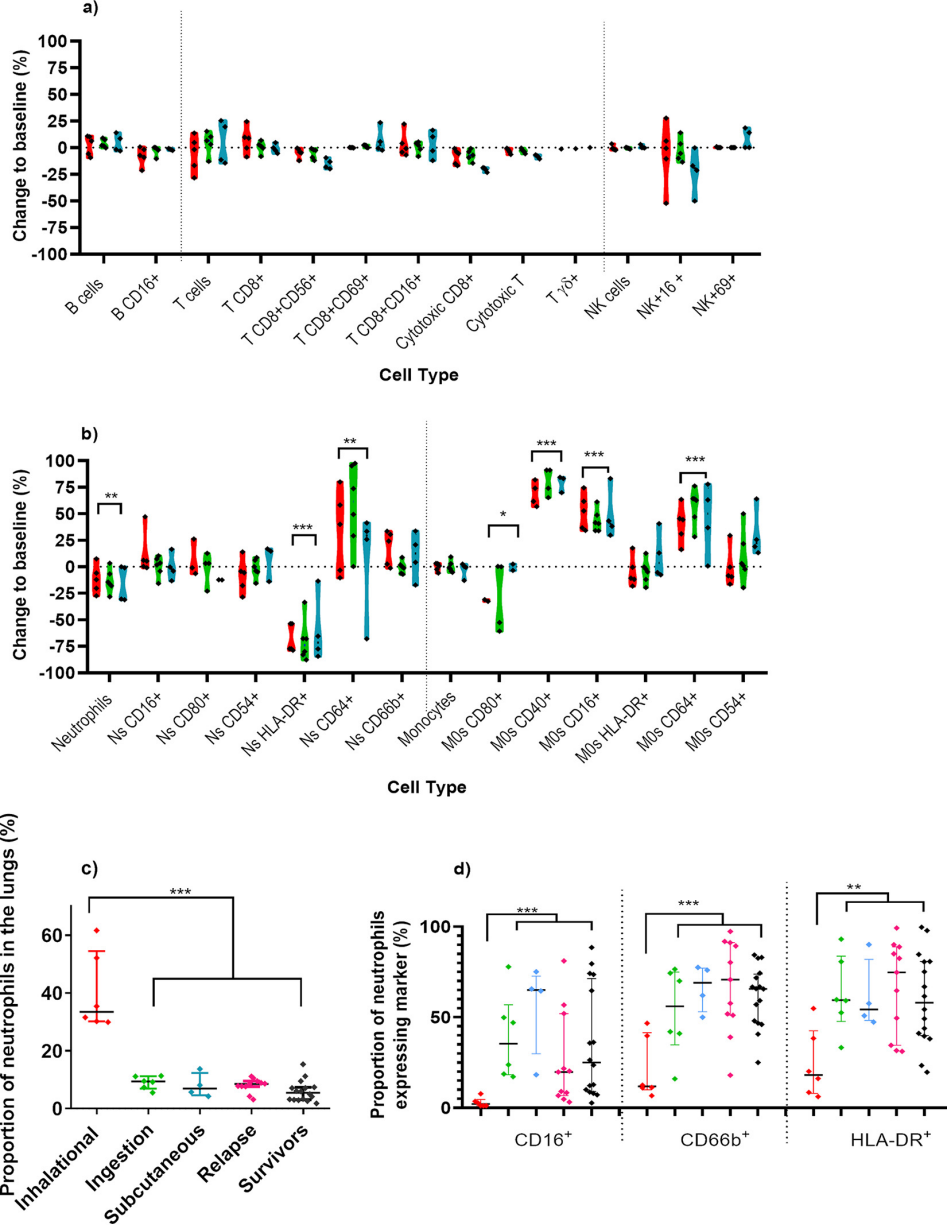
Changes in cell populations and phenotype of the immune cells in animals that received placebo and succumbed following challenge with B. pseudomallei by various routes. Lymphocyte (a) and neutrophil and monocyte (b) profiles of changes between matched blood samples were compared to baseline levels for animals challenged by the inhalational (red), ingestion (green), or subcutaneous (blue) routes. Data are presented for each individual animal and grouped by challenge routes. The proportion (c) and expression (d) of neutrophils in the lung were compared to animals that relapsed (pink) or survived until the end of the study (black). The significant difference for combined routes determined by one sample *t* test for (a) and (b) and Kruskal-Wallis ANOVA for (c) and (d), where *, *P* < 0.05; **, *P* <0.01; ***, *P* < 0.001.

Cell phenotypes characterized from blood samples obtained at the humane endpoint for the placebo-treated animals were compared to those found in the baseline samples for each individual animal (Fig. 6). Minimal changes were observed in the levels of lymphocytes; however, there were significant changes in the proportion and activation marker expression of the neutrophils and monocytes in the blood (Fig. 6b). Terminal disease was characterized by a mean reduction of 13.5% in the proportions of neutrophils (*P* = 0.0011), a mean increase of 36.2% in neutrophil expression of CD64^+^ (the sepsis marker, *P* = 0.0068), and a mean reduction of 66.8% in neutrophil expression of HLA-DR^+^ (*P* < 0.0001), regardless of the route of infection. Levels of monocytes did not change; however, there were significant increases in expression markers, with a mean increase of 75.8% in expression of CD40^+^, 47.6% in CD16^+^, and 48% in CD64^+^ (all *P* < 0.0001), whereas expression of CD80^+^ decreased by 22% (*P* = 0.043). All animals had high levels greater than 1,000 pg/mL of IFN-γ, TNF-α, IL-6, and IL-1β in the plasma (data not shown).

The proportion of neutrophils was assessed in lung homogenates from animals at the time of euthanasia. Only animals that had been challenged by the inhalational route had detectable infiltration of neutrophils into the lungs (median of 33.5%, with a range of between 29.9 and 61.6%, compared to a median of 8.5%, range 4.2 to 9.5% for other routes) (Fig. 6c; *P* < 0.0001). These neutrophils were characterized by loss of CD16^+^ and CD66b^+^ (maturity markers, both *P* < 0.001), loss of HLA-DR (health marker, *P* = 0.0011) (Fig. 6d), and to a lesser extent CD80^+^ and CD54^+^ (function). For the other routes of infection, the lung neutrophil population was similar in proportion and expression to naive animals or animals that survived challenge. There was no discernible difference in the proportions of lung macrophages according to route of infection, but there was a reduction in macrophages in the placebo animals compared to survivors (median value of 5%, range 0.6 to 9%, compared to a median of 12.5%, range 0.6 to 23.5%, *P* < 0.0001), but a mean increase of 38% in CD40^+^ expression (classical phagocytic activation) on the few present (*P* < 0.0001).

### Treatment allows the development of an immune response that controls disease in some animals.

**FIG 7:**
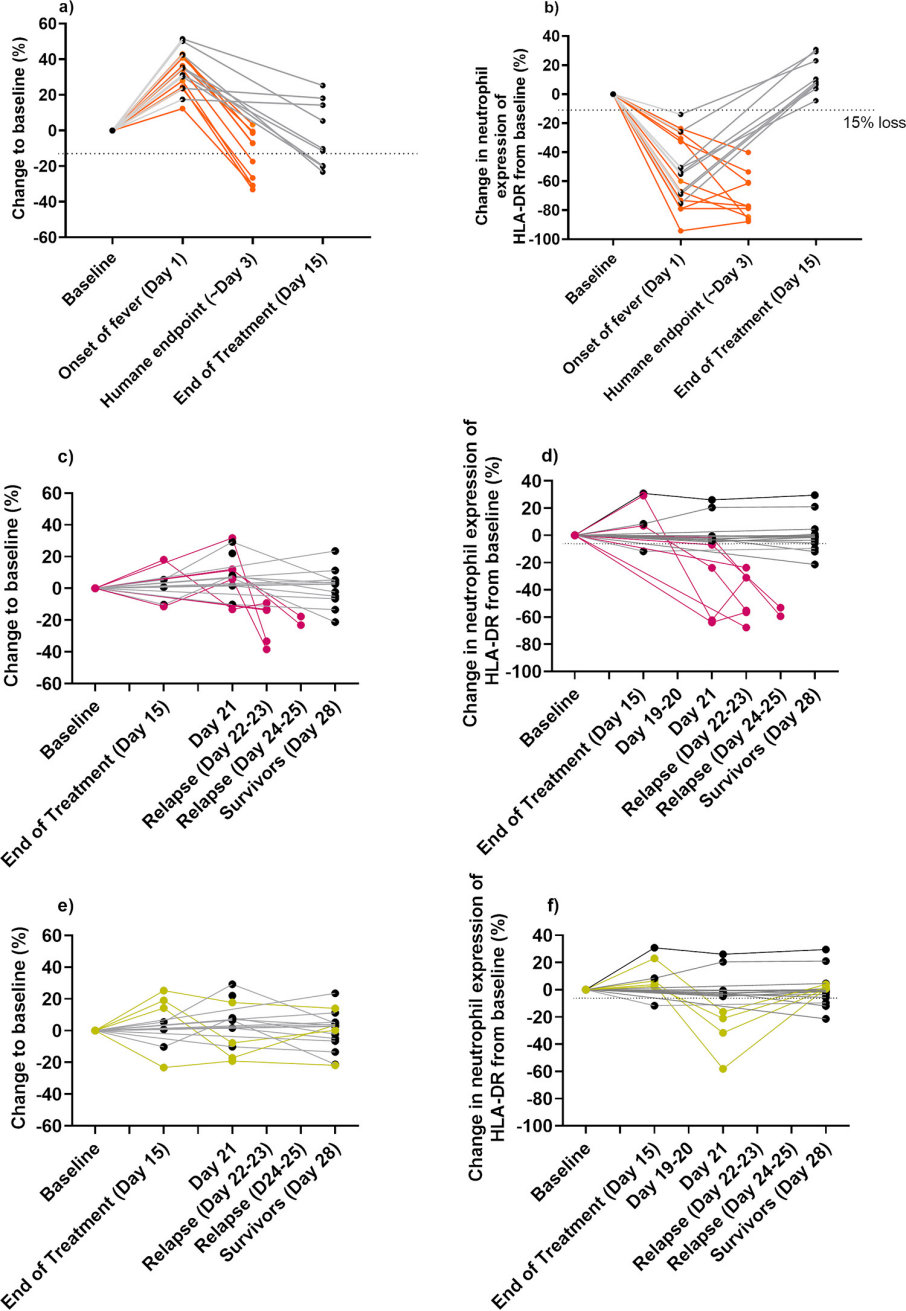
Changes in the proportions of circulating neutrophils and the associated HLA-DR expression levels following challenge with B. pseudomallei by various routes. The change in the proportion of neutrophils (a) and expression of HLA-DR^+^ (b) in animals that received placebo and succumbed to disease (orange lines). The change in the proportion of neutrophils (c) and expression of HLA-DR^+^ (d) in animals that relapsed following 14 days of treatment with co-trimoxazole and succumbed to disease (pink lines). The change in the proportion of neutrophils (e) and expression of HLA-DR^+^ (f) in animals that relapsed following 14 days of treatment with co-trimoxazole and had an immunological subclinical disease (green lines). In all cases, levels are compared to animals that survived the duration of the study (gray lines).

Levels of neutrophils and HLA-DR^+^ expression were compared for individual animals from blood samples collected during the course of the disease. At the onset of fever, the levels of neutrophils increased in all but one animal, and the expression of HLA-DR^+^ decreased in all animals compared to their individual baseline samples (Fig. 7a and b). In animals that received placebo, the HLA-DR^+^ levels continued to decrease until the animals succumbed to disease (Fig. 7b). However, the HLA-DR^+^ levels returned to normal by day 15 in all animals that received co-trimoxazole for 14 days. The expression of CD16^+^ on neutrophils had also returned to baseline levels, and circulating IFN-γ was not detected. Following the cessation of treatment, there were three outcomes: animals remained healthy and survived the duration of the study; they relapsed and succumbed to disease; or there was evidence of an immunological subclinical disease. Animals that survived maintained normal levels of neutrophils and HLA-DR^+^ expression and showed no signs of infection. Relapse in animals was preceded with a reduction in the level of that animal's HLA-DR^+^ expression, which was evident in blood taken less than 3 days prior to the animal’s succumbing (Fig. 7d). This was evident in 3 animals, which had reduced HLA-DR^+^ expression on day 21 and then succumbed by day 24. Circulating IFN-γ was also detected in blood samples taken less than 3 days prior to the animals succumbing, but not earlier. A reduction in the proportion of neutrophils was the only evidence in blood collected postmortem. All animals that succumbed had detectable circulating IFN-γ. Four animals (two challenged by the inhalational route, one by the subcutaneous route, and one by the ingestion route) whose levels of neutrophils and HLA-DR^+^ expression had returned to normal on day 15, had reduced HLA-DR^+^ expression on day 21 and detectable circulating IFN-γ but did not succumb to disease (Fig. 7e and f). These animals showed no other indication of disease, and the HLA-DR^+^ expression had returned to normal at the end of the study (day 28).

### Comparison of the immune response of animals that succumbed and those that survived.

**FIG 8:**
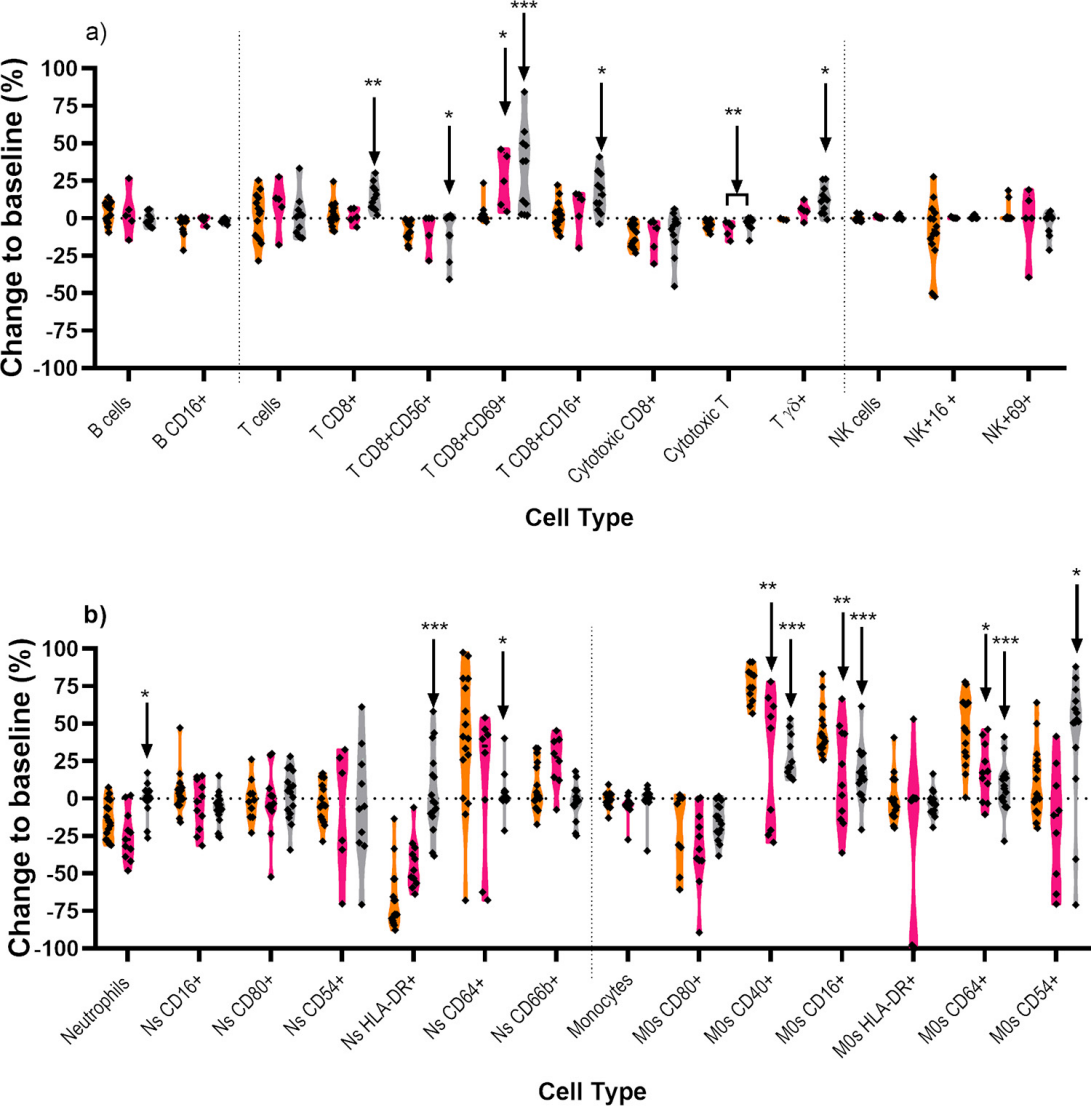
Comparison of the changes in cell populations and phenotype of different groups of animals challenged with B. pseudomallei by various routes of challenge. The changes in lymphocyte (a) and neutrophil and monocyte (b) levels of animals that received placebo (orange), relapsed following treatment with co-trimoxazole (pink), or survived following treatment with co-trimoxazole (gray) are compared to matched baseline data. Significant differences shown are between pooled placebo changes, and either pooled relapse or survival data were determined by Kurskal-Wallis ANOVA where *, *P* < 0.05; **, *P* < 0.01; ***, *P* < 0.001.

The phenotype and genotype of the immune cells in the blood were compared for animals that received placebo and succumbed to disease, those that received co-trimoxazole and relapsed, and those that survived until the end of the study (Fig. 8). Immunologically, there was little difference between animals that succumbed to disease regardless of whether this was within 3 days of challenge (placebo animals) or 16 to 22 days later (animals that relapsed following treatment). Two differences observed were the increase in the CD69^+^CD8^+^ cells (8.7% versus 21.4%, *P* = 0.011) and the decreased expression of CD40^+^ (25.1% versus 12.0%. *P* = 0.003), CD16^+^ (30.7% versus 17.0%, *P* = 0.009), and CD64^+^ (31.5% versus 18.5%, *P* = 0.013) on macrophages. Lung samples from relapsing animals did not show any infiltration of neutrophils (between 3.1 and 11.1%), regardless of the original route of infection. This is in stark contrast to the infiltration of neutrophils observed in the placebo animals challenged by the inhalational route (29.9 to 61.6%).

**FIG 9:**
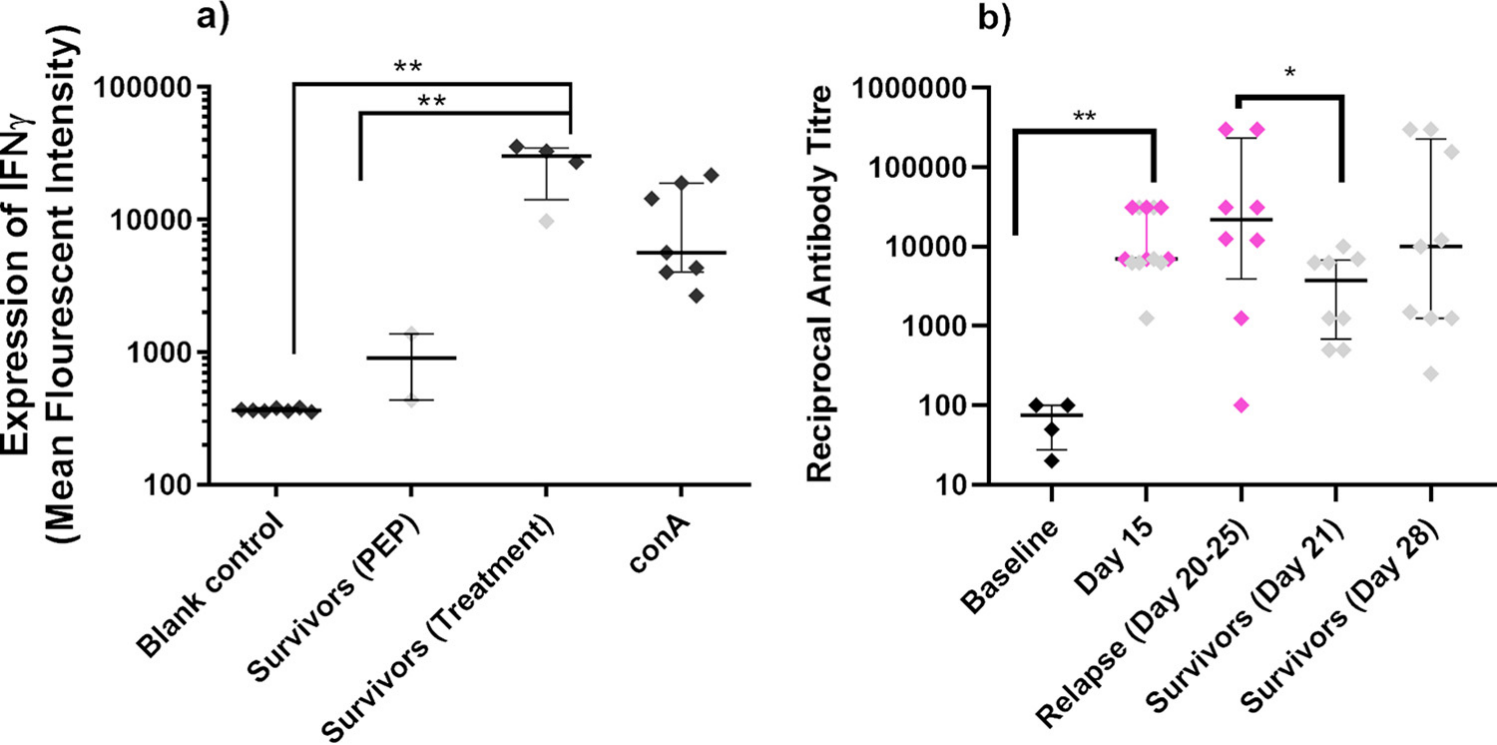
Immunological memory assessed using a cell restimulation assay (a) or detection of IgG by ELISA (b). The IFN-γ response to heat-killed B. pseudomallei from mixed splenic cell populations was measured from animals that survived following treatment with co-trimoxazole and challenge with B. pseudomallei. ELISA plates were coated with 1 × 10^8^ CFU/mL of heat-killed B. pseudomallei.

The most notable differences occurred in animals that survived until the end of the study. There was evidence of T-cell activation, by increased proportion of CD8^+^ T cells (11.7% versus 22.6%, *P* = 0.004) and reduction in CD56^+^ expression, but increase in both CD16^+^ (11.5% versus 20.7%, *P* = 0.018) and CD69^+^ (8.7% versus 21.5%, *P* = 0.0006) activity. There was significant expansion of the circulating γδ T-cell populations (3.5% versus 12.5%, *P* = 0.028). The importance of T cells in surviving animals was demonstrated in a spleen restimulation assay where significant increases in IFN-γ production were observed compared with the negative control (increased by 80-fold, *P* = 0.0042), and those from animals that had survived treatment started at 6 h postchallenge (increased by 30-fold, *P* = 0.0039) (Fig. 9a).

The B. pseudomallei-specific IgG levels were assessed at various time points postchallenge (Fig. 9b). There was significant antibody produced by day 15 (reciprocal titer of at least 7 × 10^3^, *P* = 0.005), but there was no predictive difference between the levels generated by the animals that would go on to succumb or survive. Upon relapse (days 20 to 25), 2 out of 8 animals generated high levels of antibody (>1.5 ×10^5^ reciprocal titer), which was significantly different from those that were still healthy (*P* = 0.047). However, there was no difference from the response in the 3 out of 9 healthy animals by day 28 (>1.5 × 10^5^ reciprocal titer), suggesting that antibody in this model provided little protection.

## DISCUSSION

The aim of this work was to compare the efficacy of co-trimoxazole for melioidosis acquired by different exposure routes in a single animal model. Co-trimoxazole was selected as the test antibiotic due to it being recommended for melioidosis prophylaxis and the clinical use of the drug in the eradiation phase of melioidosis following intensive intravenously therapy with either meropenem or ceftazidime (3). Marmoset models of the three naturally occurring routes of infection, cutaneous, inhalational, and ingestion, have been developed at the Defence Science and Technology Laboratory (Dstl) and were used in four independent studies (10 to 12).

The initial starting dose for co-trimoxazole in the pharmacokinetic studies was based on the human dosage and scaled in accordance with the FDA Guidance document (14). Nonparametric modeling and noncompartmental analysis of the pharmacokinetic parameters predicted that 12 hourly dosing of 13/66 mg/kg of co-trimoxazole would result in concentration of sulfamethoxazole remaining above the MIC for 99.7% of the time in a 24-h period. Additionally, the area under the curve (AUC) following a single dose was greater than the recommended target for sulfamethoxazole of 675 μg/h.mL (15). For melioidosis, time above the MIC of >60% as well as an AUC_0-24_/MIC ratio of greater than 25 typically result in successful treatment (16). The dosing regimen above meets all these parameters. However, the modeling predicted that trimethoprim concentrations would not reach the MICs following 12 hourly dosing of 13/66 mg/kg co-trimoxazole. This would require greater than 1,000-fold concentrations of co-trimoxazole, which is practically not feasible and is likely to result in toxicity. However, in human studies, trimethoprim levels are significantly greater in the tissue than the plasma (17). A plasma-to-tissue ratio of greater than 1:20 for trimethoprim and sulfamethoxazole typically exhibits therapeutic benefit. Indeed, similar plasma ratios to those observed in marmosets have a clinical benefit in melioidosis patients (16). Maintenance of levels of sulfamethoxazole alone above the MIC was sufficient to protect BALB/c mice from a lethal challenge of B. pseudomallei (8). Therefore, this regimen was used to assess the efficacy of co-trimoxazole in the melioidosis models.

Co-trimoxazole has been recommended for eradication phase treatment as well as prophylaxis for melioidosis (3). The prophylactic use of co-trimoxazole, as well as doxycycline, had previously been demonstrated as being effective as prophylaxis when administered by 6 h postchallenge (8, 9, 18). However, co-trimoxazole was significantly better when administered at 24 h postexposure compared to both doxycycline and amoxicillin-clavulanic acid (9). A small-scale study was performed to assess prophylaxis at 6 h postchallenge, with marmosets challenged by inhalational, subcutaneous, and ingestion routes of exposure. All treated animals survived for 14 days following the cessation of antibiotic, without any fever or clinical signs. No bacteria were recovered from any of the organs assessed, and only minimal physiological changes were observed, indicating clearance of the bacteria. This further demonstrates the utility of co-trimoxazole as a postexposure prophylactic. Due to the complete protection observed, further studies focused on using temperature as a trigger-to-treat.

Prior to the initial antibiotic dose, blood was collected from the majority of animals to assess bacteremia and look for immunological biomarkers of disease. Bacteraemia was only observed in 67% of animals, although all animals, except one, had an increase in the number of neutrophils and low levels of expression of neutrophils expressing HLA-DR^+^. The neutrophil expression of HLA-DR^+^ has previously been identified by our group as an important general indicator of the health status of marmosets (13). The animal that had a high/normal level of expression of HLA-DR^+^ was bacteremic. Therefore, all animals exhibited some signs of disease prior to the administration of co-trimoxazole. In humans, bacteremia is found in between 38 and 73% of patients (19). Various degrees of bacteremia have been observed in nonhuman primates (both rhesus macaques and African green monkeys), with 16 out of 20 animals having bacteria in their blood on at least one occasion between days 1 and 4 postchallenge, with animals becoming febrile between days 1 and 2 (20). An initial increase in the number of neutrophils was also observed in these animals for the first 2 days postchallenge, and similarly to the marmosets, these levels dropped with time.

All animals administered co-trimoxazole at the onset of fever (trigger-to-treat) survived the duration of treatment. However, from 4 days following the cessation of treatment, some animals relapsed. Even with intensive intravenous treatment followed by oral treatment in humans, recurrent melioidosis occurs. The reported incidence of recurrent melioidosis varies from 5.7% in one study in Darwin (21) to around 10% in East Asia (22). The use of multilocus sequence typing (MLST) has been used to distinguish between patients that relapse and those that are actually reinfected. A small study identified that only 1 in 4 cases of recurrent melioidosis in South India were due to relapse (23), whereas in a larger study in Thailand, 75% of cases were relapse (24). In murine studies, relapse was observed in 56% of BALB/c mice administered co-trimoxazole from 6 h postchallenge for 14 days (8). However, the rate of relapse decreased to 17% following treatment for 21 days. Therefore, longer treatment, or an alternative antibiotic, in the marmoset study may have decreased the incidence of relapse. Indeed, relapse in humans is linked to the duration of oral antibiotic treatment (22).

Marmosets challenged by the ingestion route were the least likely to relapse, with significantly more animals relapsing following inhalational and subcutaneous challenge. This was only apparent when treatment was initiated at the onset of fever, as all animals administered co-trimoxazole prophylactically survived regardless of the route of challenge. In clinical cases, the route of infection is often unknown, although there are some strong associations. These include a high incidence of disease associated with workers in paddy fields and with extreme weather conditions, suggesting subcutaneous and inhalational exposure, respectively (25, 26). However, a study in Australia indicates that at least 75% of cases are not associated with exposure to the soil (27). The hypothesis that ingestion is an important route of acquisition for melioidosis has gained momentum (28).

Licensure of antimicrobials for naturally occurring melioidosis is likely to be supported through clinical trials in regions of endemicity, where the majority of the disease is acquired by the cutaneous or ingestion route. However, in a biodefense or deliberate release scenario, high-dose, inhalational challenge doses are most likely. The marmoset data suggest extrapolating from clinical trials in regions of endemicity may result in a misrepresentation of the effectiveness of the medical countermeasure for inhalational challenge and hence a deliberate release. Therefore, better case history, identification of the route of infection in humans, and representative preclinical infection models are critical to support these clinical trials.

Many aspects of the disease in marmosets that relapsed following antibiotic administration were similar to those observed in placebo-control animals. This included the duration of disease, liver dysfunction, coagulopathy, presence of hepatitis, splenitis, and disseminating bacterial spread (10, 29). However, there were some distinct differences: histologically, animals that relapsed following inhalational challenge had less severe or atypical pneumonia, unusually with lesions in various parts of the gastrointestinal tract. The bacterial load profile was also distinctly different, with a higher number of bacteria in the liver, spleen, kidney, and blood and lower numbers in lungs of these animals. This indicates a potential shift away from pneumonic disease following antibiotic treatment. Additionally, lower numbers of lymphocytes were observed in the blood of animals that relapsed, irrespective of the challenge route.

Immunologically, there were no differences in the systemic response related to the route of infection in placebo-control marmosets, apart from the presence of high numbers of neutrophils in the lungs of animals following inhalational challenge. Acute melioidosis in marmosets is characterized by significant neutrophil and macrophage activation (13). However, distinct differences in the immunological response were observed between the placebo-control animals and those that relapsed or survived. Relapsed disease was as acute as the disease in placebo-control animals, characterized by the same neutrophil and macrophage activity, but there was also some evidence of T-cell activity, and a significant level of specific antibody. Animals that survived until the end of the study had very little neutrophil activity, but there was still macrophage activity, although reduced compared to acute disease in placebo-control animals. More significantly, there were considerable increases in T-cell populations (specifically, γδ T cells and CD8^+^ T cells) and their activation status, along with evidence of specific T-cell-mediated immunity and antibody. Specific T-cell activity is positively linked to survivors of acute melioidosis (30, 31). However, despite high levels of antibody found in survivors, there is only limited evidence for a protective effect from antibody in human disease (32, 33), despite the importance of vaccine-associated antibody for survival in animal studies (34, 35). The immunological evidence suggests that the marmosets’ recovery is more likely linked to greater T-cell activity (CD8^+^, CD8^+^ CD16^+^, γδ T cells, γδ^+^ CD56^+^ T cells, NK CD16^+^) than antibody levels. Interestingly, there was also immunological evidence of subclinical relapse occurring in several animals that showed no other clinical signs, which confirms the beneficial effect of extended treatment regimes.

Overall, this study demonstrated a link between the route of infection and the incidence of relapse in a suboptimal antibody treatment regimen. It also demonstrated that aspects of the disease presentation, particularly immunological, changed following antibiotic treatment, and T-cell activity was linked to survival.

## MATERIALS AND METHODS

### Biosafety committee approval.

All work with Burkholderia pseudomallei was carried out within fully contained class III microbiological safety cabinets within a BSL3 laboratory, in facilities that are registered and inspected by the UK Health and Safety Executive, as required under the Control of Substances Hazardous to Health (COSHH) regulations and all associated guidance for BSL3 pathogens. These pathogens were handled in accordance with safety documentation that was reviewed by the Biological Safety Risk Committee and the Biological Safety Officer and authorized by management.

### Animals.

Healthy, sexually mature common marmosets (Callithrix jacchus) were obtained from the Defence Science and Technology (Dstl) Porton Down breeding colony and housed in female and vasectomized male pairs. Animals were aged between 1.8 and 5 years old and weighed between 350 and 538 g at the time of study. The animals were allowed free access to food and water as well as environmental enrichment. Prior to use in challenge studies, all animals were surgically implanted intraperitoneally (i.p.), under general anesthesia (ketamine/medetomidine/isoflurane), with a Remo 201 device to record core body temperature (Tc) remotely (EMMS Bordon, Hampshire, United Kingdom). Prophylactic pain relief of 0.2 mg/kg of meloxicam and 0.005 mg/kg of buprenorphine was administered. Data were analyzed using the eDacq software to provide real-time and recordable Tc (EMMS Bordon, Hampshire, United Kingdom). At least 7 days prior to challenge, baseline blood was collected from all animals to assess baseline levels of immunological parameters (see Immunological Analysis section below). Animals were anesthetized with 5 mg of ketamine hydrochloride, a 23-gauge needle was attached to a 5-mL syringe, and up to 2.5 mL of blood was collected from the femoral vein. Blood was also collected postchallenge at the onset of fever, day 7, day 14, and day 21.

All animal studies were carried out in accordance with the UK Animals (Scientific Procedures) Act of 1986 and the Codes of Practice for the Housing and Care of Animals used in Scientific Procedures 1989. Following challenge with B. pseudomallei, all animals were handled under the UK Advisory Committee on Dangerous Pathogens (ACDP) animal containment level 3 conditions (equivalent to CDC/NIH Biosafety level 3), within a half-suit isolator compliant with British Standard BS5726.

### Determination of the pharmacokinetics of co-trimoxazole in the common marmoset.

Two studies were performed, a single-dose study and a multidose study. A 23-gauge needle was attached to a 3 mL syringe, and up to 0.5 mL of blood was collected from the femoral vein of each animal four times during a 24-h period. Blood was collected in a lithium heparin tube prior to mixing thoroughly. The blood was then centrifuged for 5 min at 13,000 rpm. Plasma was collected into a labeled, sterile Eppendorf tube and stored at −70°C until analysis. Plasma concentrations were modeled using the Phoenix WinNonLin (Pharsight v 6.1) software to determine parameters such as half-life (t_1/2_), AUC, maximum concentration of drug (Cmax), etc. Nonparametric superposition modeling was performed to assess the effect of changing the initial dose and the frequency of dosing. Noncompartmental analysis (NCA) was used to assess the time above the MIC.

**(i) Single-dose study.** A cohort of 6 animals was administered 13/66 mg/kg (79 mg/kg) of co-trimoxazole (i.e., a concentration of 13 mg/kg of trimethoprim and 66 mg/kg of sulfamethoxazole) on a single occasion by the oral route. Blood was collected from the animals in sparse random design using a randomization table, where blood was withdrawn from three randomly allocated animals at each of 0.5, 1, 2, 4, 6, 8, 12, and 24 h postdosing.

**(ii) Multidose study.** Blood was collected from a further cohort of animals after Dose 1 (day 1) and Dose 9 (day 5) in sparse random design using a randomization table, where blood was withdrawn from four randomly allocated animals at each of 0.5, 1, 2, 4, 6, and 12 h postdosing.

### Quantification of co-trimoxazole concentrations in marmoset plasma.

The levels of co-trimoxazole in plasma were determined using liquid chromatography–tandem mass spectrometry (LC-MS). Sulphamethoxazole and trimethoprim were individually weighed and dissolved in dimethyl sulfoxide (DMSO) to give 50 mg/mL (sulfamethoxazole) and 5 mg/mL (trimethoprim) stock solutions. Dextrometorphan (DXT) was dissolved in DMSO to give a 1-mg/mL stock solution and diluted to 1 μg/mL with acetonitrile containing 2.5% DMSO to give the working internal standard (IS) solution. Marmoset plasma standard (50 μL) or sample was mixed with 150 μL of the IS solution and centrifuged, and the supernatant decanted into a 250-μL HPLC polypropylene vial. The supernatants were reduced in volume using a Genevac centrifugal evaporator for 90 min at 40°C. Twenty microliters of 0.1% formic acid were added and mixed by vortex before being injected onto the LC-MS system (Agilent 1100, CTC PAL, Sciex 3000). Calibration curves were produced over the range 50 to 50,000 μg/mL for sulfamethoxazole and 5 to 1,000 ng/mL for trimethoprim.

### Bacterial strain and culture.

Glycerol stocks of B. pseudomallei strain K96243 were provided by Battelle Biomedical Research Centre. A frozen stock of B. pseudomallei was thawed and streaked onto Luria-Bertani supplemented with 5% glycerol (LBG) agar plates. The plates were incubated at 37^º^C for between 23.5 and 25.3 h (mean time of 23.9 ± 0.27 h). A loopful of the colonies was used to inoculate approximately 5 mL of phosphate-buffered saline (PBS), and the optical density (OD_600_) determined (CO7500 Colorimeter, WPA Colourwave). The OD_600_ was adjusted to between 0.35 and 0.38, which is equivalent to approximately 1 × 10^8^ CFU/mL. One mL of final suspension was used to inoculate 100 mL LBG broth. The broth cultures were incubated at 37^º^C on a rotary shaker (Innova 4200 Shaking Incubator, New Brunswick Scientific) at 180 rpm for a between 15 and 16.9 h (mean time of 16.3 ± 0.27 h). The broth was removed from the shaker and the OD_600_ measured in triplicate using 1 mL aliquots in a cuvette. The OD_600_ of 1 mL of the 100 mL culture was adjusted with PBS to between 0.35 and 0.38, which is equivalent to approximately 1 × 10^8^ CFU/mL. A series of 10-fold dilutions (10^−1^ to 10^−8^) was prepared by aliquoting 1 mL of the OD-adjusted culture into 9 mL of PBS. The appropriate dilution was selected for challenge. In parallel, the dilution series was used to determine the viable count by aliquoting a single 250-μL aliquot of each of the 10^−5^, 10^−6^, 10^−7^, and 10^−8^ dilution onto four separate LBG agar plates. Plates were incubated at 37 ^º^C for at least 24 h prior to enumeration.

### Challenge.

For ingestion challenge, 100 μL of the 1 × 10^8^ CFU/mL suspension of bacteria (neat OD-adjusted culture) was added to 1 mL of banana-favored Nesquik powder dissolved in water. The liquid was presented in a syringe to preconditioned animals to accept. For subcutaneous challenge, 100 μL of the 1 × 10^3^ CFU/mL suspension of bacteria (10^−5^ dilution of the neat OD-adjusted culture) was injected into a pinch of skin fold in the inner thigh of the animals.

Inhalational challenge was performed using a contained Henderson apparatus controlled by the AeroMP (Aerosol Management Platform) aerosol system (Biaera Technologies LLC). The aerosol was generated using a 3-jet Collison nebulizer into the piccolo tube. Animals were removed from their home cages and sedated with 10 mg of ketamine hydrochloride by the intramuscular route. Once the animals were unresponsive to ocular and pedal stimulation, they were placed within a plethysmography tube and attached to the exposure unit. Two animals were exposed to bacteria per aerosol run for 10 min. The aerosol cloud was sampled at the midway point of challenge (4 min 30 s to 5 min 30) into PBS via an All-Glass Impinger (AGI-30; Ace Glass, Vineland, NJ). An Aerodynamic Particle Sizer Spectrometer sampled the aerosol cloud for 1 min before and after the collection of the impinger sample, and the median mass aerodynamic diameter (MMAD) and geometric standard deviation (GSD) of the bacterial particles were determined. The accumulated volume of air breathed by each animal was determined by real-time plethysmography using eDacq software (Version 1.8.4b).

The challenge dose was determined following the enumeration of the impinger concentration and calculated as follows:

$$\mathrm{Aerosol}\;\mathrm{concentration}\;\left(\text{CFU}/\text{L of air}\right)=\frac{\text{impinger count }\left(\text{CFU}/\text{mL}\right)\text{ × impinger volume }\left(\text{mL}\right)}{\text{impinger flow rate }\left(L/\text{minute}\right)\;\text{× impinger time }\left(\text{minutes}\right)}$$

$$\;\mathrm{Dose}\;\mathrm{received}\;\left(\mathrm{CFU}\right)=\text{ aerosol concentration }\left(\text{CFU}/\text{L of air}\right)\text{ × total accumulated volume }\left(L\right)$$

### Antibiotic dosing schedule.

All animals receiving antibiotic were given 79 mg/kg of co-trimoxazole at 12 hourly intervals in banana-favored Nesquik powder dissolved in water. This was dispensed in a syringe orally to individual animals on 28 occasions. Placebo-control animals received banana-favored Nesquik powder dissolved in water for the same duration. Animals were monitored for a period up to 21 days after antibiotic dosing was stopped. Animals were culled on reaching the humane endpoint or at the scheduled end of the study.

### Bacteriological analysis.

Bacterial loads were determined in blood, liver, spleen, kidneys, and lungs. Organs were removed aseptically and processed as previously described (36). Appropriate dilutions were subcultured onto LB-agar plates for B. pseudomallei and incubated at 37°C for 24 h. Counts are expressed as CFU.g^−1^ of tissue or CFU.mL^−1^ of blood.

### Immunological analysis.

Single-cell suspensions of blood (postred cell lysis) collected pre- and postchallenge, and lung collect at the time of euthanasia points, were stained using fluorescent anti-human antibody sets to identify cell phenotypes and activation status by flow cytometry. Antibodies used were the following: for lymphocytes, CD3^+^ (SP34-2), CD8^+^ (LT8), CD56^+^ (B159), CD69^+^ (FN50), TCRγδ^+^ (B1), CD20^+^ (Bly1), and CD16^+^ (3G8); for monocytes/macrophages and neutrophils, CD11c^+^ (SHCL3), CD14^+^ (M5E2), CD16^+^ (3G8), CD40^+^ (5C3), CD80^+^ (2D10), CD54^+^ (HCD54), CD163^+^ (GHI/61), and HLA-DR^+^ (L243) (BD Bioscience, BioLegend, AbD Serotec). Stained cells were fixed for 48 h in a final volume of 4% paraformaldehyde. Samples were analyzed on a BD FACSCanto II and cell populations determined using BD FACSDiva software. Whole cells (as determined by the presence of an intact nucleus but not necessarily an intact cell membrane) were detected by nuclear staining, allowing the area of interest to be defined by forward and side scatter. Forward and side scatter were also used to gate areas for detection of lymphocytes (T and B cells), natural killer cells (NK), macrophages (M0), and neutrophils.

### Restimulation assay.

Single-cell suspensions of spleen cells were diluted to achieve an estimated density of 1 to 3 × 10^6^ cells/mL, and stimulated with either L-15 media (negative control), ConA (2.5 μg/mL positive control; Sigma), or 1 × 10^7^ CFU/mL of heat-killed B. pseudomallei for 18 h incubated at 37°C. The supernatant was removed and stored at −80°C prior to cytokine analysis.

### Antibody ELISA.

Plasma was stored frozen until analysis. Maxisorb 96-well plates were coated overnight with 1 × 10^8^/mL of heat-killed B. pseudomallei in carbonate coating buffer, and blocked for 2 h with bovine serum albumin (BSA). Plasma, at a starting dilution of 1 in 50, and further dilutions of 1 in 5, was allowed to bind for 1 h. Antibody binding was detected with a goat anti-human IgG horseradish peroxidase (HRP) after a further hour.

### Cytokine analysis.

Cytokines and chemokines were measured in the plasma, which were stored frozen at −80°C until required. Levels of cytokines and chemokines were quantified using the human flexset for IL-1β, IL-6, MCP-1, and RANTES (BD cytokine flex beads) and for TNF-α and IFN−γ (reagents from U-CyTech Biosciences and Mabtech AB, conjugated to flex beads by BBI Detection, Ltd.). All samples were fixed in 4% paraformaldehyde for 36 h at 4°C and analyzed by flow cytometry (FACS Canto II BD).

### Histopathological analysis.

Tissues were fixed in 10% neutral buffered formalin and processed for paraffin wax embedding using standard techniques. Thin sections (4 μm) were cut and stained with hematoxylin and eosin (H&E) for histopathological analysis. A scoring system based on the type of lesions and organ distribution was established in order to allow semiquantitative comparison (29).

### Immunohistochemistry (IHC).

IHC staining was performed on selected tissues (liver, spleen, lungs, GI tract) for the detection of bacterial antigen, T-cells (CD3^+^), macrophages, and neutrophils (MAC387^+^). A semiquantitative scoring system was established in order to allow comparison (29). The avidin biotin complex (ABC, Vector Elite; Vector laboratories) method was used for immunolabelling. Four-micrometer sections of formalin-fixed wax-embedded tissues were prepared and dried onto polylysine slides (VWR, Ltd.). The sections were dewaxed, rehydrated, and then treated in hydrogen peroxide 3% (vol/vol), then in methanol for 15 min to eliminate endogenous peroxidase activity. The tissue sections were then pretreated for antigen retrieval by enzymatic digestion with either pronase XIV (0.05% wt/vol pronase XIV 5.2U/mg; Sigma P5147-1G) or trypsin/alpha-chymotrypsin (0.5% trypsin and 0.5% alpha chymotrypsin; Sigma-Aldrich, Gillingham, Dorset, United Kingdom) at 37°C for 10 min. The sections were then microwaved in citric acid buffer (2.1 g citric acid [Fisher Scientific, Loughborough, Leicestershire, United Kingdom] in 1,000 mL distilled water, pH 6.0) for 18 min at 100°C, 90% effect (780W), or microwaved in Dako high pH 9.0 buffer for 10 min. The sections were then mounted in a Sequenza Immunostaining Centre (Shandon Scientific, Runcorn, United Kingdom) and rinsed with Tris-buffered saline (TBS) pH 7.6, 0.005 M (Sigma-Aldrich, USA). Primary antibody cross-reactivity with tissue constituents was prevented using 1.5% normal serum block applied to the sections for 20 min. The serum block matched the host species in which the link antibody was raised. Details of primary antibodies used, specificity, concentration, and incubation time are summarized in Table 2. All primary antibodies had been previously screened to determine the optimum dilution and incubation temperature. The sections were washed in TBS and then incubated for 30 min with the appropriate biotinylated secondary link antibody (Vector Laboratories) before being washed twice in TBS again. The sections were incubated for 30 min at room temperature with Avidin Biotin complex (Vector Elite kit, Vector Laboratories), and the signal was detected using 3,30-diaminobenzidine tetrahydrochloride (DAB). Finally, the sections were lightly counterstained with Mayer’s hematoxylin (Surgipath, Peterborough, United Kingdom) for 5 min, dehydrated in absolute alcohol, and cleared in xylene before being coverslipped. These included sequential sections with an isotype control for each primary antibody, and the omission of the primary antibody.

### Statistics.

Pearson’s correlation analysis was used to determine the relationship of gender, body weight, time to death, and inhaled dose. Comparative analysis of bacteriology, immunology, and blood chemistry data were performed using two-way ANOVA. Survival data were analyzed using a log rank (Mantel-Cox) test.

## References

[1] Wiersinga WJ, Virk HS, Torres AG, Currie BJ, Peacock SJ, Dance DAB, Limmathurotsakul D. 2018. Melioidosis. Nat Rev Dis Primers 4:22. 10.1038/nrdp.2017.107. 29388572 PMC6456913

[2] Limmathurotsakul D, Kanoksil M, Wuthiekanun V, Kitphati R, deStavola B, Day NPJ, Peacock SJ. 2013. activities of daily living associated with acquisition of melioidosis in Northeast Thailand: a matched case-control study. PLoS Negl Trop Dis 7:e2072. 10.1371/journal.pntd.0002072. 23437412 PMC3578767

[3] Lipsitz R, Garges S, Aurigemma R, Baccam P, Blaney DD, Cheng AC, Currie BJ, Dance D, Gee JE, Larsen J, Limmathurotsakul D, Morrow MG, Norton R, O’Mara E, Peacock SJ, Pesik N, Rogers LP, Schweizer HP, Steinmetz I, Tan G, Tan P, Wiersinga WJ, Wuthiekanun V, Smith TL. 2012. Workshop on treatment of and postexposure prophylaxis for *Burkholderia pseudomallei* and *B. mallei* infection, 2010. Emerg Infect Dis 18:e2. 10.3201/eid1812.120638. PMC3557896 23171644

[4] Dance D. 2014. Treatment and prophylaxis of melioidosis. Int J Antimicrob Agents 43:310–318. 10.1016/j.ijantimicag.2014.01.005. 24613038 PMC4236584

[5] Chetchotisakd P, Chierakul W, Chaowagul W, Anunnatsiri S, Phimda K, Mootsikapun P, Chaisuksant S, Pilaikul J, Thinkhamrop B, Phiphitaporn S, Susaengrat W, Toondee C, Wongrattanacheewin S, Wuthiekanun V, Chantratita N, Thaipadungpanit J, Day NP, Limmathurotsakul D, Peacock SJ. 2014. Trimethoprim-sulfamethoxazole versus trimethoprim-sulfamethoxazole plus doxycycline as oral eradicative treatment for melioidosis (MERTH): a multicentre, double-blind, non-inferiority, randomised controlled trial. Lancet 383:807–814. 10.1016/S0140-6736(13)61951-0. 24284287 PMC3939931

[6] Majoni SW, Hughes JT, Heron B, Currie BJ. 2018. Trimethoprim+sulfamethoxazole reduces rates of melioidosis in high-risk hemodialysis patients. Kidney Int Rep 3:160–167. 10.1016/j.ekir.2017.09.005. 29340327 PMC5762962

[7] Chau KWT, Smith S, Kang K, Dheda S, Hanson J. 2018. Antibiotic prophylaxis for melioidosis in patients receiving hemodialysis in the tropics? One size does not fit all. Am J Trop Med Hyg 99:597–600. 10.4269/ajtmh.18-0421. 30014827 PMC6169155

[8] Barnes KB, Steward J, Thwaite JE, Lever MS, Davies CH, Armstrong SJ, Laws TR, Roughley N, Harding SV, Atkins TP, Simpson AJH, Atkins HS. 2013. Trimethoprim/sulfamethoxazole (co-trimoxazole) prophylaxis is effective against acute murine inhalational melioidosis and glanders. Int J Antimicrob Agents 41:552–557. 10.1016/j.ijantimicag.2013.02.007. 23517714

[9] Sivalingam SP, Sim SH, Jasper LC, Wang D, Liu Y, Ooi EE. 2008. Pre- and post-exposure prophylaxis of experimental *Burkholderia pseudomallei* infection with doxycycline, amoxicillin/clavulanic acid and co-trimoxazole. J Antimicrob Chemother 61:674–678. 10.1093/jac/dkm527. 18192684

[10] Nelson M, Dean RE, Salguero FJ, Taylor C, Pearce PC, Simpson AJH, Lever MS. 2011. Development of an acute model of inhalational melioidosis in the common marmoset (*Callithrix jacchus*). Int J Exp Pathol 92:428–435. 10.1111/j.1365-2613.2011.00791.x. 22122591 PMC3248079

[11] Nelson M, Salguero FJ, Dean RE, Ngugi SA, Smither SJ, Atkins TP, Lever MS. 2014. Comparative experimental subcutaneous glanders and melioidosis in the common marmoset (*Callithrix jacchus*). Int J Exp Pathol 95:378–391. 10.1111/iep.12105. 25477002 PMC4285464

[12] Nelson M, Nunez A, Ngugi SA, Atkins TP. 2021. The lymphatic system as a potential mechanism of spread of melioidosis following ingestion of *Burkholderia pseudomallei*. PLoS Negl Trop Dis 15:e0009016. 10.1371/journal.pntd.0009016. 33617546 PMC7932547

[13] Ngugi S, Laws T, Simpson AJ, Nelson M. 2022. The innate immune response in the marmoset during the acute pneumonic disease caused by *Burkholderia pseudomallei*. Infect Immun 90:e00550-21. 10.1128/iai.00550-21. PMC8929355 35041487

[14] Food and Drug Administration. 2005. Estimating the maximum safe starting dose in initial clinical trials for therapeutics in adult healthy volunteers—guidance for industry. FDA, Rockville, MD.

[15] van der Ven AJ, Mantel MA, Vree TB, Koopmans PP, van der Meer JW. 1994. Formation and elimination of sulphamethoxazole hydroxylamine after oral administration of sulphamethoxazole. Br J Clin Pharmacol 38:147–150. 10.1111/j.1365-2125.1994.tb04339.x. 7981016 PMC1364861

[16] Cheng AC, McBryde ES, Wuthiekanun V, Chierakul W, Amornchai P, Day NPJ, White NJ, Peacock SJ. 2009. Dosing regimens of cotrimoxazole (trimethoprim-sulfamethoxazole) for melioidosis. Antimicrob Agents Chemother 53:4193–4199. 10.1128/AAC.01301-08. 19620336 PMC2764189

[17] Wormser GP, Keusch GT, Heel RC. 1982. Co-trimoxazole (trimethoprim-sulfamethoxazole)—an updated review of its anti-bacterial activity and clinical efficacy. Drugs 24:459–518. 10.2165/00003495-198224060-00002. 6759092

[18] Gelhaus HC, Anderson MS, Fisher DA, Flavin MT, Xu ZQ, Sanford DC. 2013. Efficacy of post exposure administration of doxycycline in a murine model of inhalational melioidosis. Sci Rep 3:1146. 10.1038/srep01146. 23359492 PMC3556592

[19] Gassiep I, Ganeshalingam V, Chatfield MD, Harris PNA, Norton RE. 2022. Melioidosis: laboratory investigations and association with patient outcomes. Am J Trop Med Hyg 106:54–59. 10.4269/ajtmh.21-0548. PMC8733490 34724627

[20] Yeager JJ, Facemire P, Dabisch PA, Robinson CG, Nyakiti D, Beck K, Baker R, Pitt ML. 2012. Natural history of inhalation melioidosis in rhesus macaques (*Macaca mulatta*) and African green monkeys (*Chlorocebus aethiops*). Infect Immun 80:3332–3340. 10.1128/IAI.00675-12. 22778104 PMC3418741

[21] Sarovich DS, Ward L, Price EP, Mayo M, Pitman MC, Baird RW, Currie BJ. 2014. Recurrent melioidosis in the Darwin Prospective Melioidosis Study: improving therapies mean that relapse cases are now rare. J Clin Microbiol 52:650–653. 10.1128/JCM.02239-13. 24478504 PMC3911345

[22] Limmathurotsakul D, Chaowagul W, Chantratita N, Wuthiekanun V, Biaklang M, Tumapa S, White NJ, Day NP, Peacock SJ. 2008. A simple scoring system to differentiate between relapse and re-infection in patients with recurrent melioidosis. PLoS Negl Trop Dis 2:e327. 10.1371/journal.pntd.0000327. 18958279 PMC2570249

[23] Halim I, Shaw T, Tellapragada C, Vandana KE, Mukhopadhyay C. 2017. Melioidosis: reinfection going incognito as relapse. Indian J Med Microbiol 35:593–596. 10.4103/ijmm.IJMM_17_140. 29405156

[24] Limmathurotsakul D, Chaowagul W, Chierakul W, Stepniewska K, Maharjan B, Wuthiekanun V, White NJ, Day NP, Peacock SJ. 2006. Risk factors for recurrent melioidosis in northeast Thailand. Clin Infect Dis 43:979–986. 10.1086/507632. 16983608

[25] Cheng AC, Jacups SP, Gal D, Mayo M, Currie BJ. 2006. Extreme weather events and environmental contamination are associated with case-clusters of melioidosis in the Northern Territory of Australia. Int J Epidemiol 35:323–329. 10.1093/ije/dyi271. 16326823

[26] Dance DAB. 1991. *Pseudomonas-pseudomallei*: danger in the paddy fields. Trans R Soc Trop Med Hyg 85:1–3. 10.1016/0035-9203(91)90134-K. 2068733

[27] Merianos A, Patel M, Lane JM, Noonan CN, Sharrock D, Mock PA, Currie B. 1993. The 1990–1991 outbreak of melioidosis in the Northern Territory of Australia: epidemiology and environmental studies. Southeast Asian J Trop Med Public Health 24:425–435. 7512752

[28] Limmathurotsakul D, Peacock SJ. 2011. Melioidosis: a clinical overview. Br Med Bull 99:125–139. 10.1093/bmb/ldr007. 21558159

[29] Nelson M, Nunez A, Ngugi SA, Sinclair A, Atkins TP. 2015. Characterization of lesion formation in marmosets following inhalational challenge with different strains of *Burkholderia pseudomallei*. Int J Exp Pathol 96:414–426. 10.1111/iep.12161. 26852689 PMC4744822

[30] Jenjaroen K, Chumseng S, Sumonwiriya M, Ariyaprasert P, Chantratita N, Sunyakumthorn P, Hongsuwan M, Wuthiekanun V, Fletcher HA, Teparrukkul P, Limmathurotsakul D, Day NP, Dunachie SJ. 2015. T-cell responses are associated with survival in acute melioidosis patients. PLoS Negl Trop Dis 9:e0004152. 10.1371/journal.pntd.0004152. 26495852 PMC4619742

[31] Nithichanon A, Rinchai D, Buddhisa S, Saenmuang P, Kewcharoenwong C, Kessler B, Khaenam P, Chetchotisakd P, Maillere B, Robinson J, Reynolds CJ, Boyton RJ, Altmann DM, Lertmemongkolchai G. 2018. Immune control of *Burkholderia pseudomallei*—common, high-frequency T-cell responses to a broad repertoire of immunoprevalent epitopes. Front Immunol 9:484. 10.3389/fimmu.2018.00484. 29616023 PMC5869189

[32] Chaichana P, Jenjaroen K, Chumseng S, Sumonwiriya M, Rongkard P, Kronsteiner B, Teparrukkul P, Limmathurotsakul D, Day NPJ, Chantratita N, Dunachie SJ. 2021. Role of *Burkholderia pseudomallei*-specific IgG2 in adults with acute melioidosis, Thailand. Emerg Infect Dis 27:463–470. 10.3201/eid2702.200213. 33496230 PMC7853568

[33] Chaichana P, Kronsteiner B, Rongkard P, Teparrukkul P, Limmathurotsakul D, Chantratita N, Day NPJ, Fletcher HA, Dunachie SJ. 2020. Serum from melioidosis survivors diminished intracellular *Burkholderia pseudomallei* growth in macrophages: a brief research report. Front Cell Infect Microbiol 10:442. 10.3389/fcimb.2020.00442. 32984070 PMC7479196

[34] Burtnick MN, Shaffer TL, Ross BN, Muruato LA, Sbrana E, DeShazer D, Torres AG, Brett PJ. 2018. Development of subunit vaccines that provide high-level protection and sterilizing immunity against acute inhalational melioidosis. Infect Immun 86:e00724-17. 10.1128/IAI.00724-17. 29109172 PMC5736816

[35] Khakhum N, Bharaj P, Myers JN, Tapia D, Kilgore PB, Ross BN, Walker DH, Endsley JJ, Torres AG. 2019. *Burkholderia pseudomallei* ΔtonB Δhcp1 live attenuated vaccine strain elicits full protective immunity against aerosolized melioidosis infection. mSphere 4:e00570-18. 10.1128/mSphere.00570-18. 30602524 PMC6315081

[36] Nelson M, Lever MS, Savage VL, Salguero FJ, Pearce PC, Stevens DJ, Simpson AJH. 2009. Establishment of lethal inhalational infection with *Francisella tularensis* (tularaemia) in the common marmoset (*Callithrix jacchus)*. Int J Exp Pathol 90:109–118. 10.1111/j.1365-2613.2008.00631.x. 19335549 PMC2676706

